# Synthetic pathways for microbial biosynthesis of valuable pyrazine derivatives using genetically modified *Pseudomonas putida* KT2440

**DOI:** 10.1016/j.mec.2025.e00258

**Published:** 2025-03-30

**Authors:** Vytautas Petkevičius, Justė Juknevičiūtė, Domas Mašonis, Rolandas Meškys

**Affiliations:** Department of Molecular Microbiology and Biotechnology, Institute of Biochemistry, Life Sciences Center, Vilnius University, Saulėtekio 7, Vilnius, LT-10257, Lithuania

**Keywords:** 2,5-Dimethylpyrazine, 5-Methyl-2-pyrazinecarboxylic acid, 2,5-Dimethylpyrazine 1-oxide, 2,5-Dimethylpyrazine 1,4-dioxide, Non-heme diiron monooxygenase, *Pseudomonas putida* KT2440

## Abstract

Using engineered microbes for synthesizing high-valued chemicals from renewable sources is a foundation in synthetic biology, however, it is still in its early stages. Here, we present peculiarities and troubleshooting of the construction of novel synthetic metabolic pathways in genetically modified work-horse *Pseudomonas putida* KT2440. The combination of this microbial host and heterologous expressed non-heme diiron monooxygenases enabled *de novo* biosynthesis of 2,5-dimethylpyrazine (2,5-DMP) carboxylic acid and *N*-oxides as target products. A key intermediate, 2,5-DMP, was obtained by using *Pseudomonas putida* KT2440Δ6 strain containing six gene deletions in the L-threonine pathway, along with the overexpression of *thrA*^*S345F*^ and *tdh* from *E. coli*. Thus, the carbon surplus was redirected from glucose through L-threonine metabolism toward the formation of 2,5-DMP, resulting in a product titre of 106 ± 30 mg L^−1^. By introducing two native genes (*thrB* and *thrC* from *P. putida* KT2440) from the L-threonine biosynthesis pathway, the production of 2,5-DMP was increased to 168 ± 20 mg L^−1^. The resulting 2,5-DMP was further derivatized through two separate pathways. Recombinant *P. putida* KT2440 strain harboring xylene monooxygenase (XMO) produced 5-methyl-2-pyrazinecarboxylic acid from glucose as a targeted compound in a product titre of 204 ± 24 mg L^−1^. The microbial host containing genes of PmlABCDEF monooxygenase (Pml) biosynthesized *N*-oxides – 2,5-dimethylpyrazine 1-oxide as a main product, and 2,5-dimethylpyrazine 1,4-dioxide as a minor product, reaching product titres of 82 ± 8 mg L^−1^ and 11 ± 2 mg L^−1^ respectively.

## Highlights

• *P. putida* KT2440 was engineered for the biosynthesis of pyrazine derivatives.
• A novel biosynthesis pathway of MCPA from glucose using a single microbial host.
• A first report of biosynthetic pathways to obtain 2,5-dimethylpyrazine *N*-oxides.
• Non-heme diiron monooxygenases Pml and XMO are utilized in metabolic engineering.

## 1 Introduction

2,5-Dimethylpyrazine (2,5-DMP) is a valuable alkylpyrazine contributing to the flavor of cocoa beans, peanuts, and soybean-based fermented foods (Ren et al., 2024; Mortzfeld et al., 2020). Due to its aromatic properties, 2,5-DMP is an attractive natural flavor additive for the food industry (Fayek et al., 2023). Moreover, derivatives of 2,5-DMP are key intermediates in the synthesis of certain pharmaceutical products and other important compounds (Huigens et al., 2022; Hou et al., 2023). For instance, 5-methylpyrazine-2-carboxylic acid (MPCA) can be produced from 2,5-DMP via oxidation of one of the methyl groups (Fang et al., 2018). MPCA plays a crucial role in synthesizing commercial drugs such as acipimox (a lipid-lowering agent), glipizide (an anti-diabetic medication), pyrazinamide compounds (medications used to treat tuberculosis), and many more (Gu et al., 2020). The oxidation of 2,5-DMP can also yield *N*-oxides, including 2,5-dimethylpyrazine 1-oxide (2,5-DMP-N-OX), which facilitates complex transformations due to its enhanced reactivity and versatility (Clayden et al., 2012). It is used to synthesize various alkyl-substituted pyrazines including aroma compounds, pheromones, and pharmacologically active agents (Souza et al., 2023; Ghosh et al., 2019). In addition, 2,5-dimethylpyrazine 1,4-dioxide (2,5-DMP-di-OX) exhibits similar activation properties to its mono-N-oxide analogue and is particularly valuable for synthesis of novel polymer building block compounds such as pyrazine-fused isoindigos (Li et al., 2017), self-assembling homoleptic polymers (Donaldson et al., 2024), or used by itself as a ligand in coordination polymers (Mishra et al., 2024).

Currently, 2,5-DMP and its derivatives are primarily produced through chemical synthesis. The Maillard reaction and Strecker degradation are the main methods to obtain 2,5-DMP (Amrani-Hemaimi et al., 1995; Yaylayan, 2003), while oxidation routes facilitate the production MPCA, 2,5-DMP-N-OX, and 2,5-DMP-di-OX. Alternatively, biocatalytic approaches leveraging synthetic metabolic pathways offer more environmentally friendly solutions. However, research on metabolic engineering for pyrazine derivatives remains limited. For example, the biosynthesis of *N*-oxides mostly covers the synthesis of phenazine *N*-oxides through intermediates of the shikimate pathway, utilizing the engineered strain *Pseudomonas chlororaphis* HT66 (Guo et al., 2020). Additionally, nonribosomal peptide synthetases-mediated conversions of amino acids, such as valine, lead to the production of valdiazen and various 2,5-diisopropylpyrazine *N*-oxides (Kretsch et al., 2018; Morgan and Li, 2020). These processes rely on existing metabolic pathways, with no reports of *de novo* biosynthetic design for 2,5-DMP *N*-oxides. MCPA can be synthesized via the whole-cell transformation of 2,5-DMP using *Pseudomonas putida* strain (Kiener, 1992). A biosynthetic pathway has also been described, though it requires three different recombinant microbial hosts and complex collaborative fermentation (Feng et al., 2020), highlighting the need for a simpler, more efficient approach. Metabolic engineering efforts have been more successful for 2,5-DMP production. Recently, several studies have demonstrated 2,5-DMP synthesis through the L-threonine synthesis pathway in *E. coli* (Xu et al., 2020; Yang et al., 2021; Liu et al., 2023, 2024; Zeng et al., 2024). However, in previous reports, 2,5-DMP has been regarded primarily as the final product of the biosynthesis pathways. Its potential role as an intermediate for producing more diverse pyrazine compounds, through the new metabolic pathway extensions, is unknown. Exploring new metabolic routes could unlock its potential for broader applications. Therefore, advanced strategies such as the engineering and utilizing different microbial strains and diverse enzymes to obtain pyrazine derivatives should be pursued.

In this work, we challenge the established microbial production strategies of pyrazine derivatives in *E. coli* and highlight the potential of *P. putida* KT2440 as a novel host for pyrazine biosynthesis via the unique application of non-heme diiron monooxygenases Pml and XMO. Our findings have shown that (1) 2,5-DMP biosynthesis is achievable in the KT2440 strain, (2) the biosynthesis of MPCA from glucose is feasible in shake flask cultivation using a single microbial host, (3) 2,5-DMP-N-OX and 2,5-DMP-di-OX can be produced via *de novo* biosynthesis.

## 2 Results

### 2.1 Designing a strategy for the production of 2,5-dimethylpyrazine derivatives in *P*. *putida* KT2440

**Fig. 1:**
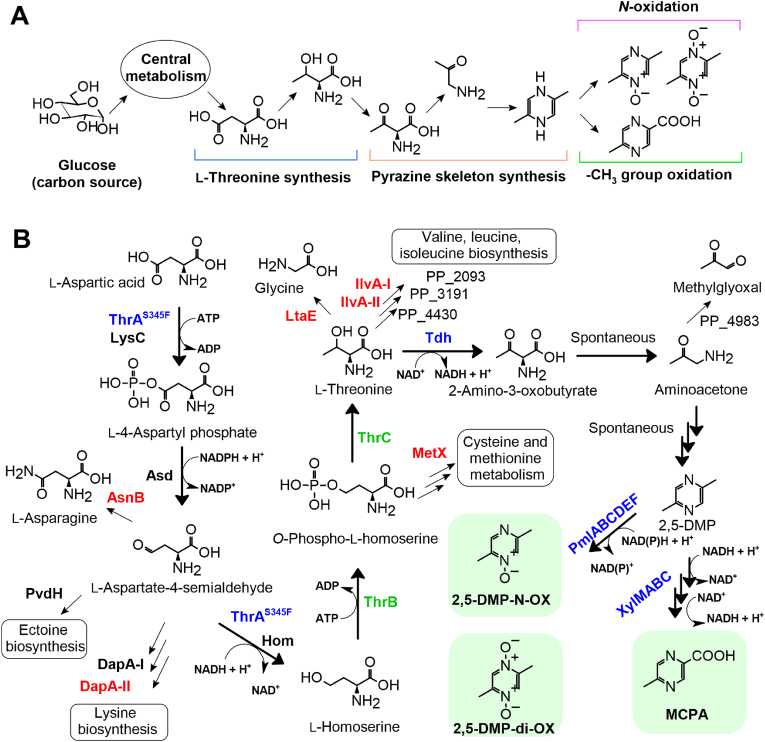
A strategy for the development of biosynthetic pathways leading to 2,5-DMP and its derivatives. A) General scheme of pyrazine derivative biosynthesis from glucose through central metabolism. B) A detailed synthesis pipeline highlighting enzymes of interest in the pyrazine derivatives-producing strain *P. putida* KT2440Δ6: those responsible for product synthesis are shown in black (native), green (homologous overexpressed) and blue (heterologous overexpressed), while those leading to side reactions (of which appropriate coding genes have been deleted) are shown in red. Targeted products are depicted in green rectangles.

L-threonine metabolism functions as a primary pathway for producing the pyrazine skeleton structure, as it is the most straightforward method that has consistently proven to be effective (Zhang et al., 2020; Liu and Quan, 2024). Threonine dehydrogenase (Tdh) catalyzes the oxidation of the hydroxyl group in threonine, producing 2-amino-3-oxobutyrate. This intermediate undergoes spontaneous reactions, culminating in the formation of 2,5-DMP, a central metabolite from which multiple products can be derived. For the *N*-oxidation reactions, we selected the non-heme diiron monooxygenase PmlABCDEF (Petkevičius et al., 2019), whereas the oxidation leading to MCPA was assigned to a different non-heme diiron monooxygenase, XMO (Kiener, 1992), encoded by the *xylMABC* cluster (Fig. 1A). Both pathways require robust L-threonine production from glucose, ensuring efficient 2,5-DMP formation in host cells Thus, carbon flux from glucose must be channeled through an enhanced threonine biosynthesis pathway, seamlessly integrated with pyrazine production. To implement this concept, we executed genetic modifications with a few additional adjustments: (i) maintaining a streamlined metabolic engineering approach, leveraging well-characterized enzymes; and (ii) accounting for previous studies in *E. coli*, adjusting for potential differences in *P*. *putida* KT2440. Thus, based on the KEGG database and available literature, we proposed the engineered metabolic pathway encompassing necessary genes for pyrazine biosynthesis (Fig. 1B).

In *P*. *putida* KT2440, the primary precursor of L-threonine is L-aspartate, derived from glucose via central carbon metabolism. L-Aspartate is enzymatically converted to L-threonine through a cascade involving aspartate kinase (encoded by *lysC*), aspartate semialdehyde dehydrogenase (encoded by *asd*), homoserine dehydrogenase (encoded by *hom*), homoserine kinase (encoded by *thrB*), and threonine synthase (encoded by *thrC*). To bridge L-threonine synthesis with the pathway of pyrazine derivatives, the addition of recombinant threonine dehydrogenase is required, as *P*. *putida* KT2440 lacks this gene. However, the proposed biosynthesis pathway includes competing reactions that may negatively impact pyrazine production. L-Aspartate, for instance, serves as a synthesis precursor for asparagine (catalyzed by asparagine synthetase, gene *asnB* (PP_1750)), and L-aspartate-4-semialdehyde, which is utilized in lysine biosynthesis by 4-hydroxy-tetrahydrodipicolinate synthases (coded by *dapA-I* (PP_1237) and *dapA-II* (PP_2639) respectively) and in L-2,4-diaminobutanoate synthesis via diaminobutyrate-2-oxoglutarate transaminase (coded by *pvdH,* (PP_2800, PP_4223)). Furthermore, homoserine serves as a substrate for homoserine acetyltransferase (encoded by *metX* (PP_5097)), which is involved in the metabolism of methionine and cysteine, while L-threonine itself can be diverted toward valine, leucine, and isoleucine synthesis, catalyzed by threonine deaminases (*ilvA-I* (PP_3446) and *ilvA-II* (PP_5149)). Additionally, L-threonine can be converted to glycine via threonine aldolase (encoded by *ltaE* (PP_0321)). It is also possible that certain putative enzymes, which were not considered as candidates in this study – such as L-serine dehydratase (PP_2093), threonine ammonia-lyase/dehydratase (PP_3191), threonine dehydratase (PP_4430), and amine oxidase (PP_4983) – may also contribute to the consumption of intermediates. Overall, our proposed scheme identifies key genes essential for precursor and product synthesis while highlighting those that divert flux away from the desired pathway and should be suppressed.

### 2.2 Designing microbial chassis for L-threonine and 2,5-DMP production

**Fig. 2:**
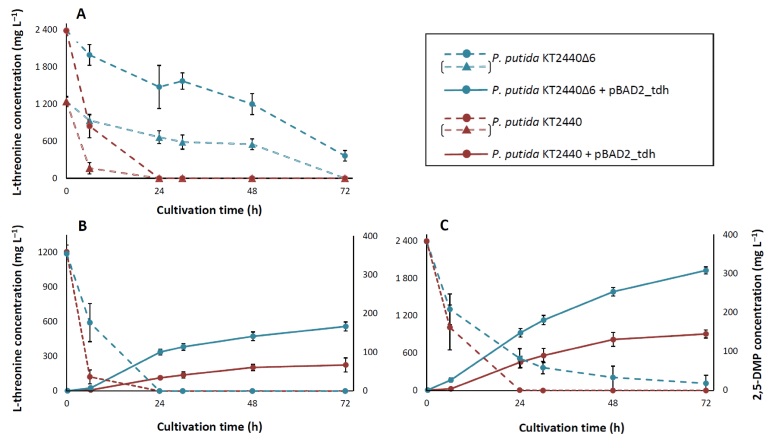
A time-course of L-threonine consumption and 2,5-DMP synthesis. A) L-threonine depletion by the *P. putida* KT2440 (brown lines) and KT2440Δ6 (blue lines). Hollowed lines depict a starting concentration of 1.19 g L^−1^ of threonine, full-bodied lines designate 2.38 g L^−1^ of threonine. B) Conversions by the *P. putida* KT2440 (brown lines) and KT2440Δ6 (blue lines) strains with 1.19 g L^−1^ of threonine and carrying pBAD2_tdh_plasmid; C) Conversions by the *P. putida* KT2440 (brown lines) and KT2440Δ6 (blue lines) strains with 2.38 g L^−1^ of threonine and carrying pBAD2_tdh_plasmid. Biomass concentration of 1.5 g L^−1^ CDW was used in all samples. The data represent mean values and standard deviations obtained from at least three independent bioconversions.

Our primary objective was to engineer microbial host capable of maintaining elevated levels of the key precursor L-threonine by minimizing its depletion through side enzymatic reactions. The aspartate-threonine pathway, a major metabolic route for amino acid biosynthesis during the growth phase, significantly drains the intracellular L-threonine pool (Li et al., 2017). To counteract this, we prioritized the marker-less deletion of genes responsible for the branches that lead to the biosynthesis of asparagine, lysine, cysteine, methionine, valine, leucine, isoleucine, and glycine. Through sequential deletions, we constructed a modified strain, *P. putida* KT2440Δ6, with marker-less deletions of *ltaE*, *asnB, dapA-II, ilvA-I*, *ilvA-II,* and *metX* (Fig. S1). Our initial goal also involved deletions of *pvdH* and *dapA-*I genes. Although we were unable to screen mutants containing these deletions, the resulting bacterial strain proved to be a promising chassis for our purpose. Notably, *P. putida* KT2440Δ6 exhibited significant superiority over the wild-type *P. putida* KT2440 in terms of retaining L-threonine in the reaction mixture (Fig. 2A). In a 20 mM potassium phosphate buffer (pH 7.0) containing 1.5 g L^−1^ CDW of wild-type *P. putida* KT2440, all the supplemented L-threonine (1190 mg L^−1^ and 2380 mg L^−1^, corresponding to 10 mM and 20 mM) was fully consumed within 24 h. This was confirmed by the absence of a signal in the HPLC-MS chromatogram corresponding to the extracted ion count of the L-threonine standard (120 m/z [M+H]^+^). In contrast, the L-threonine concentration in appropriate samples of *P. putida* KT2440Δ6 suspension remained relatively high after 24 h, 665 ± 105 mg L^−1^ and 1475 ± 347 mg L^−1^ respectively which is approximately half of the initial amount. Even after 72 h of incubation, detectable amounts of L-threonine persisted, unlike in the parental strain.

The next step involved integrating a suitable threonine dehydrogenase (Tdh) to connect the L-threonine pathway with the synthesis of the pyrazine skeleton. Since *P. putida* KT2440 lacks the *tdh* gene in its genome, an additional heterologous gene was introduced. We chose Tdh from *E. coli* DH5α because previous studies have shown that this enzyme was one of the most active among the various threonine dehydrogenases tested (Xu et al., 2020). The *tdh* gene was amplified by PCR and cloned into pBAD2-MCS-1 and expression vector to yield plasmid pBAD2_tdh. This plasmid was then introduced into both *P. putida* KT2440 and KT2440Δ6 followed by the whole-cell transformations with L-threonine in a potassium phosphate buffer solution (20 mM, pH = 7.0) at 30 °C. Both recombinant strains converted substrate as the synthesis of 2,5-DMP was evident (Fig. 2BC) using two different initial concentrations of L-threonine (as before, 1190 mg L^−1^ and 2380 mg L^−1^). However, the accumulation of 2,5-DMP in the parental strain plateaued after 48 h. Consequently, product titres of 76 ± 18 mg L^−1^ and 144 ± 10 mg L^−1^ were achieved using 1190 mg L^−1^ and 2380 mg L^−1^ mM of L-threonine, respectively. In contrast, the production of 2,5-DMP in KT2440Δ6 was higher and sustained for a longer period in both samples (L-threonine at 1190 mg L^−1^ and 2380 mg L^−1^), reaching titers of 166 ± 12 mg L^−1^ and 308 ± 9 mg L^−1^ after 72 h, respectively (Supplementary material, Chromatograms S5–S8). This equates to an average L-threonine–to–2,5-DMP conversion yield of 34 % by the engineered strain, significantly outperforming the 14 % yield of the wild type strain. These results align with previous findings that the *P. putida* KT2440 rapidly depletes L-threonine, reducing its availability for 2,5-DMP biosynthesis. This highlights KT2440Δ6 as a superior host for metabolic engineering strategies aimed at L-threonine overproduction. Additionally, the Tdh enzyme from *E. coli* DH5α proved to be a proper biocatalyst for 2,5-DMP production.

### 2.3 Increasing biosynthesis of L-threonine and 2,5-DMP in KT2440Δ6

**Fig. 3:**
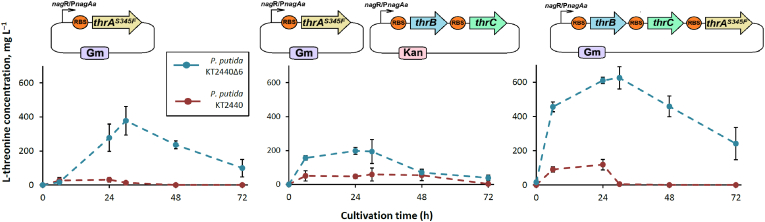
A time-course of L-threonine biosynthesis in *P. putida* KT2440 and KT2440Δ6 strains using various plasmid combinations. A) pJNN_thrA^S345F^; B) pJNN_thrA^S345F^ and pBNT_thrB_thrC; C) pJNN _thrB_thrC_thrA^S345F^. The graphs illustrate L-threonine biosynthesis by the KT2440Δ6 (blue lines) and *P. putida* KT2440 (brown lines) strains in the MSM medium (volume of 20 mL, glucose 5.0 g L^−1^ as the main carbon and energy substrate). The induction was initiated at 0 h. The data represent mean values and standard deviations obtained from at least three independent cultivations.

The next step was to test whether the engineered strain KT2440Δ6 could not only retain but also accumulate surplus amounts of L-threonine when grown in a mineral salt medium (MSM) supplemented with glucose as the primary carbon and energy source and amino acids (lysine 0.4 g L^−1^, methionine 0.2 g L^−1^, isoleucine 0.2 g L^−1^, and asparagine 0.2 g L^−1^), whose biosynthesis pathways have been modified. L-Threonine production was analyzed with HPLC-MS and compared to the parental KT2440 strain to determine any difference. However, no trace of L-threonine was found in either sample during different cultivation stages (Supplementary material, Chromatograms S1 and S2). Similarly, neither the KT2440Δ6 strain nor the parental strain transformed with the tdh_pBAD2 plasmid showed any traces of the anticipated 2,5-DMP product when grown in MSM medium (Supplementary material, Chromatograms S3 and S4). It was evident that the KT2440Δ6 strain alone could not support 2,5-DMP formation, requiring the introduction and overexpression of genes essential for L-threonine biosynthesis. Based on our retrosynthesis analysis, those genes included aspartate kinase (encoded by *lysC*), aspartate semialdehyde dehydrogenase (*asd*), homoserine dehydrogenase (*hom*), homoserine kinase (*thrB*), and threonine synthase (*thrC*). However, first we had to address the potential feedback inhibition issue. Despite the successful overproduction of two key enzymes, aspartate kinase (LysC) and homoserine dehydrogenase (Hom) from different plasmid combinations (Supplementary material, Figs. S2 and S3), there was no increase in L-threonine production as these enzymes are known for their allosteric control (Wang et al., 2024). One way to overcome this inhibition is by using enzymes with mutations in their allosteric sites that makes them feedback-resistant. Reports suggest using LysC^T311I^, Hom^V59A^ of *Corynebacterium glutamicum*, or the bifunctional enzyme ThrA^S345F^ from *E. coli* (Li et al., 2017), which encompasses the enzymatic activities of both aspartate kinase and homoserine dehydrogenase. In this study, we used feedback-resistant ThrA^S345F^ from *E. coli*, a single enzyme capable of complementing the first three native biosynthesis enzymes (LysC, Asd, and Hom), thereby streamlining the metabolic engineering. The ThrA^S345F^ enzyme was heterologously expressed in both the KT2440Δ6 and the parental strain, resulting in soluble recombinant proteins (Supplementary material, Fig. S5). Cultivations were performed in MSM, with samples collected at various time points. The mutant strain, transformed with plasmid pJNN_thrA^S345F^, successfully produced threonine as confirmed by HPLC-MS, with synthesis peaking after 30 h and resulting in production titre of 378 ± 80 mg L^−1^ (Fig. 3). In contrast, the wild type strain harboring the same plasmid, pJNN_thrA^S345F^, yielded only traces of threonine (peaking after 24 h with 31 ± 13 mg L^−1^), underscoring the crucial role of both the ThrA^S345F^ and the KT2440Δ6 strain in this biosynthesis pathway. To further enhance L-threonine synthesis, the remaining genes of the metabolic pathway – *thrB* and *thrC* – were overexpressed as well. Two different strategies we tested: (i) single plasmid carrying all three genes (*thrA*^S345F^, *thrB* and *thrC*); and (ii) two separate plasmids (pJNN_thrA^S345F^ and pBNT_thrB_thrC). The single-plasmid approach significantly boosted threonine synthesis, yielding 625 ± 65 mg L^−1^ after 30 h of cultivation. However, expressing *thrB* and *thrC* on a separate plasmid (pBNT_thrB_thrC) had minimal impact, yielding only 195 ± 68 mg/L after 30 h and this was comparable to that of cells harboring pJNN_thrA^S345F^ alone. Nevertheless, both engineered strains outperformed the parental KT2440 strains carrying equivalent constructs (119 ± 22 mg L^−1^ and 59 ± 38 mg L^−1^, respectively). Also, despite the detectable formation of L-threonine in the wild-type strain harboring recombinant plasmids, it was rapidly consumed; after 30-48 h of cultivation, there were only traces of the product or none at all. In contrast, L-threonine biosynthesis in the mutant strain peaked after 30 h and it was still apparent after 72 h of incubation in all samples, whereas pJNN_thrA^S345F^_thrB_thrC plasmid-containing cells exhibited 242 ± 92 mg L^−1^ as the highest productivity after 72 h. Thus, the KT2440Δ6 strain proved superior to the unmodified counterpart for engineering synthetic pathways for pyrazine derivatives via L-threonine metabolism. Consequently, only the mutant strain was used in subsequent experiments for 2,5-DMP production and functionalization.

**Fig. 4:**
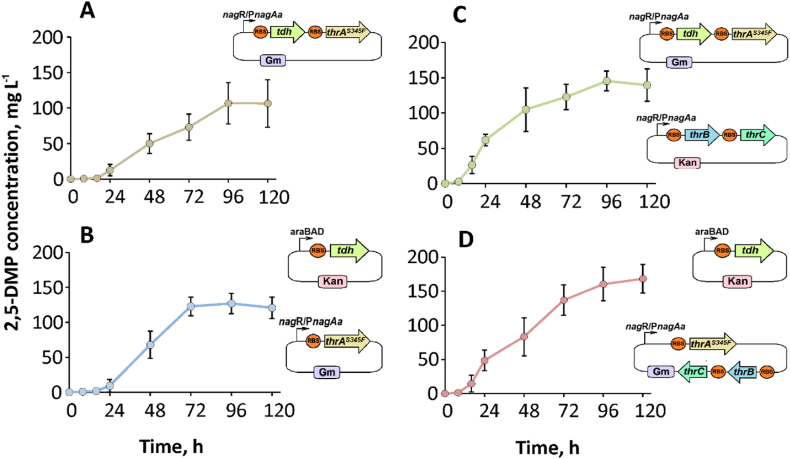
2,5-DMP biosynthesis in *P. putida* KT2440Δ6 strain. Plasmid combinations were as follows: A) pJNN_tdh_thrA^S345F^; B) pBAD2_tdh and pJNN_thrA^S345F^; C) pJNN_tdh_thrA^S345F^ with pBNT_thrB_thrC; D) pBAD2_tdh with pJNN _thrB_thrC_thrA^S345F^. The time point 0 h (0 h) indicates the stage of cultivation when induction was initiated. Cultivation was executed in MSM medium (volume of 20 mL, glucose 5.0 g L^−1^ as the main carbon and energy substrate). The data represent mean values and standard deviations obtained from at least three independent cultivations.

Next, we examined which of the generated L-threonine producers, when paired with threonine dehydrogenase (Tdh), yielded the highest levels of 2,5-DMP. To achieve this, we tested several different gene combinations. The simplest requirement for 2,5-DMP formation combines Tdh with ThrA^S345F^. Thus, we evaluated two configurations: co-expression of both genes on a single plasmid (pJNN_tdh_thrA^S345F^), and the genes expressed from separate plasmids (pBAD2_tdh and pJNN_thrA^S345F^). At the other end of the spectrum, we assessed the full biosynthetic pathway, incorporating ThrA^S345F^, ThrB, ThrC, and Tdh. Two different plasmid configurations were tested based on the combinations used in the L-threonine overproduction: pJNN_tdh_thrA^S345F^ plasmid combined with pBNT_thrB_thrC; pBAD2_tdh plasmid with pJNN_thrA^S345F^_thrB_thrC construct. All four combinations were introduced into the KT2440Δ6 strain followed by biomass cultivation, gene induction, sampling, and product analysis (Fig. 4). In all cases an accumulation of 2,5-DMP was observed.

The simplest approach – using the KT2440Δ6 strain with the pJNN_tdh_thrA^S345F^ plasmid – resulted in a production of 106 ± 30 mg L^−1^ of 2,5-DMP after 120 h of cultivation. This establishes baseline strategy for obtaining 2,5-DMP and provides an easy method for further to functionalization. Expressing *tdh* and *thrA*^*S345F*^ separately on two plasmids yielded comparable results, reaching a product titre of 121 ± 15 mg L^−1^ after 120 h. The addition of ThrB and ThrC enzymes to the biosynthesis pathway (in the form of the pBNT_thrB_thrC plasmid) had a limited impact, resulting in 140 ± 19 mg L^−1^ of pyrazine. However, the combination of pBAD2_tdh with pJNN_thrA^S345F^_thrB_thrC produced 168 ± 20 mg L^−1^ of 2,5-DMP, which was the best result among all variations tested (Supplementary material, Chromatograms S9–S12). This indicates that the formation of surplus L-threonine helped to increase 2,5-DMP production in the KT2440Δ6 strain via an engineered metabolic pathway, though gene expression from different plasmid combinations may have an impact on overall productivity.

### 2.4 Pathways of 2,5-DMP functionalization leading to *N*-oxides and carboxylic acid products

**Fig. 5:**
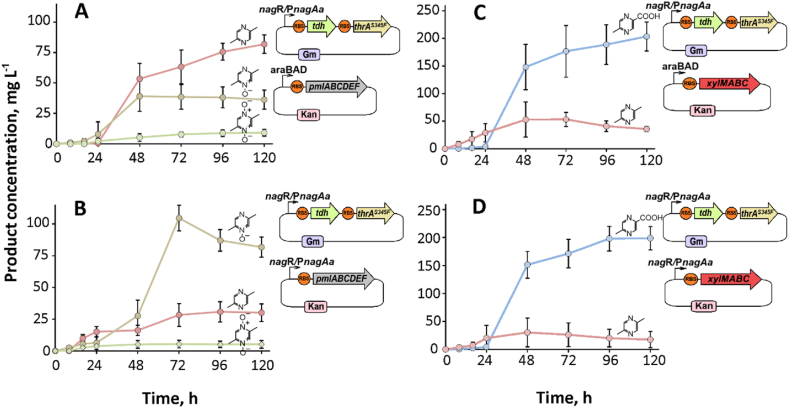
Biosynthesis of different pyrazine products in *P. putida* KT2440Δ6 strain. Plasmid combinations were as follows: A) pJNN_tdh_thrA^S345F^ and pBAD_Pml; B) pJNN_tdh_thrA^S345F^ and pBNT_Pml; C) pJNN_tdh_thrA^S345F^ and pBAD_XylMABC; D) pJNN_tdh_thrA^S345F^ and pBNT_XylMABC. The biosynthesis of various products is illustrated by the different color curves: MPCA (blue), 2,5-DMP (red), 2,5-DMP-N-OX (brown), and 2,5-DMP-di-OX (green). The induction was initiated at 0 h. Cultivation was executed in MSM medium (volume of 20 mL, glucose 5.0 g L^−1^ as the main carbon and energy substrate). The data represent mean values and standard deviations obtained from at least three independent cultivations.

Although we demonstrated that various gene combinations influence 2,5-DMP production, the recombinant strain KT2440Δ6 carrying the pJNN_tdh_thrA^S345F^ plasmid emerged as a streamlined minimal module for this process. Its simplicity and capacity to accommodate additional plasmids made this strain ideal host for functionalization experiments. To explore 2,5-DMP modifications, we introduced two monooxygenases. One of them, PmlABCDEF monooxygenase (Pml for short), converts 2,5-DMP to the *N*-oxide derivatives – 2,5-DMP-N-OX and 2,5-DMP-di-OX (Petkevičius et al., 2019). As in previous studies, we assessed the impact of *pmlABCDEF* gene expression by testing different plasmid combinations. The plasmid pBNT_Pml was used to transform a 2,5-DMP production module since it is compatible with the same inducer (sodium salicylate). Meanwhile, an alternative construct, pBAD2_Pml, was inducible with L-arabinose, allowing independent control of 2,5-DMP biosynthesis and oxidation. Both variants facilitated *N*-oxide production in a mineral salt medium (Fig. 5). Initially, the accumulation of 2,5-DMP was detected in both cases after approximately 16 h of incubation. The formation of 2,5-DMP was then followed by Pml-catalyzed oxidation to *N*-oxide products, as both 2,5-DMP-N-OX and 2,5-DMP-di-OX began to appear in HPLC chromatograms. Synthesis remained active for around 120 h until product formation plateaued. In the case of pJNN_tdh_thrA^S345F^ + pBAD2_Pml (Fig. 5A), the final product distribution after 120 h was 82 ± 8 mg L^−1^ (2,5-DMP), 30 ± 7 mg L^−1^ (2,5-DMP-N-OX), and 6 ± 2 mg L^−1^ (2,5-DMP-di-OX) when induction of both plasmids was initiated simultaneously. By contrast, the pBNT_Pml + pJNN_tdh_thrA^S345F^ transformant resulted in the higher formation of pyrazine *N*-oxides (Fig. 5B). Consequently, product titres of 36 ± 8 mg L^−1^ (2,5-DMP), 82 ± 8 mg L^−1^ (2,5-DMP-N-OX), and 11 ± 2 mg L^−1^ (2,5-DMP-di-OX) were reached after 120 h of cultivation. The varying product titres can be linked to the features of the plasmid vectors (different copy numbers, promoter strength, etc.) that translate to different levels of Pml biosynthesis. However, the residual 2,5-DMP indicated that the Pml monooxygenase is not operating properly since our previous works have shown that pyrazines can be converted at significantly higher concentrations (Petkevičius et al., 2019, 2021). To enhance *N*-oxide product synthesis, we tried several different induction and cultivation strategies, including early-phase induction and delayed induction at varying time points, but observed no significant improvement (Supplementary material, Chromatograms S13, S14). This highlights the need for more precise gene expression control to further optimize biosynthesis.

**Fig. 6:**
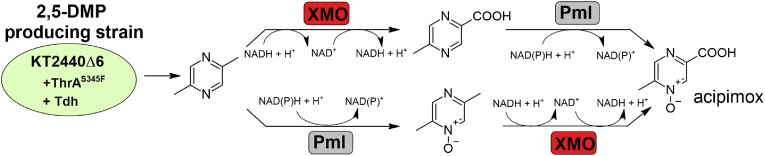
Possible metabolic pathways leading to 5-methylpyrazine-2-carboxylic acid-4-oxide (acipimox) utilizing PmlABCDEF (Pml) and XylMABC (XMO) monooxygenases.

We also studied the 2,5-DMP functionalization pathway leading to MPCA production via the utilization of XylMABC monooxygenase system (XMO for short). XMO comprises three key enzymes: XylMA hydroxylase (NADH-consuming), XylB benzyl alcohol dehydrogenase (NADH-consuming), and XylC benzaldehyde dehydrogenase (NAD^+^-consuming). Similar to Pml, the 2,5-DMP production module was transformed by combinations of plasmid pBAD2_XylMABC and pBNT_XylMABC, and pyrazine product distribution was analyzed (Fig. 6). In this instance, both plasmid variants facilitated the formation of MPCA, yielding similar amounts of product after 120 h of cultivation. Specifically, the pBAD2_XylMABC construct produced an MPCA titer of 204 ± 24 mg L^−1^, while the pBNT_XylMABC construct achieved a titer of 198 ± 21 mg L^−1^. After 120 h, the synthesis of MCPA reached a plateau, leaving intermediate compound 2,5-DMP at concentrations of 36 ± 4 mg L^−1^ and 19 ± 11 mg L^−1^ when using pBAD2_XylMABC and pBNT_XylMABC, respectively. This indicates that XMO did not perform a complete conversion under the given conditions. Therefore, we varied the induction time, as gene expression in the combination pJNN_tdh_thrA^S345F^ and pBAD2_XylMABC can be initiated separately. However, similarly to Pml, this approach did not result in any significant increase in product yield or intermediate depletion (Supplementary material, Chromatograms S15, S16), suggesting other options, reviewed in the discussion section, should be considered for future studies.

We also considered merging *N*-oxidation and methyl group oxidation pathways in pursuit of potentially new pyrazine products. However, Pml exhibits certain limitations when applied to substrates containing carboxy groups (Petkevičius et al., 2021). Consequently, the bioconversion of MPCA using pBAD2_Pml did not yield any new products (Supplementary material, Chromatogram S17). Additionally, XMO struggled to convert 2,5-DMP N-oxides; 2,5-DMP-di-OX was not transformed at all, while 2,5-DMP-N-OX was only partially converted. HPLC-MS analysis of the 2,5-DMP-N-OX reaction mixture with pBAD_XylMABC whole cells revealed the formation of a new compound with a mass increase of 16 Da, possibly indicating the presence of 2-hydroxymethyl-5-methylpyrazine-4-oxide (Supplementary material, Chromatogram S18), although its structure has not been confirmed. This suggests that even though the oxygenase XylMA converts 2,5-DMP-N-OX, the resulting alcohol product is not recognized by the *xylB*-encoded benzyl alcohol dehydrogenase to yield the corresponding acid product. Although this pathway holds promise for further development, an effective pyrazine production process using both monooxygenases (XMO and Pml) would likely require advancements in enzyme engineering.

## 3 Discussion

*Pseudomonas putida* KT2440 has evolved from a simple soil-dwelling, root-colonizing bacterium with a versatile metabolism into one of the most popular and standardized chassis for developing effective whole-cell catalysts (de Lorenzo et al., 2024). This interest stems from its robust redox metabolism, tolerance to a wide range of physicochemical stresses, and the extensive availability of molecular tools for its genetic and metabolic engineering (Martínez-García and de Lorenzo, 2024). In this work, we challenge the established microbial production strategies of 2,5-DMP in *E. coli* and highlight the potential of *P. putida* KT2440 as a host for pyrazine biosynthesis. The expansion of the 2,5-DMP metabolic pathway through the application of non-heme diiron monooxygenases, Pml and XMO, broadens the range of pyrazine products achievable through biosynthesis. Given that both enzymes originate from *Pseudomonas* spp., it is more rational to develop biocatalytic systems within compatible *Pseudomonas* hosts rather than adapting these enzymes to less suitable microorganisms. This can be perfectly illustrated by the study on MPCA biosynthesis in *E. coli* strains (Feng et al., 2020). The idea of a single *E. coli* strain harboring the entire complex enzymatic pathway had to be reconsidered due to the diverse and incompatible requirements for the heterologous biosynthesis of numerous enzymes, including XMO. As a result, the biosynthetic modules were constructed in separate cells, the process required a complex three-stage batch cultivation, involving the use of three different *E. coli* strains: one for L-threonine production, another for 2,5-DMP heterocycle formation, and a third for MCPA production employing XMO monooxygenase. This approach utilized a total of eleven genes to encode synthetic enzymes, transporters, and cofactor-regenerating enzymes, which were recruited for overexpression in chromosomes or plasmid vectors. By contrast, we demonstrated that switching to *P*. *putida* KT2440 as a host can immensely simplify MCPA biosynthesis. The derived strain KT2440Δ6 required only four additional enzymes, delivered through two separate plasmids, to produce MCPA. To our knowledge, this is the first report of MCPA biosynthesis from glucose in a single microbial host through shake flask batch cultivation. Furthermore, our work represents a significant milestone in enabling the successful use of non-heme diiron monooxygenases in metabolic engineering. These enzymes are often challenging to utilize due to differing growth and biocatalysis conditions (Staijen et al., 1997; Meyer et al., 2005; Groves et al., 2023). This challenge is particularly evident in *N*-oxide product synthesis, where previous studies have either employed a bioconversion approach (whole-cell cultivation with supplementary substrate) or relied on existing biosynthetic pathways (Mitsukura et al., 2013; Kretsch et al., 2018; Guo et al., 2020; Morgan and Li, 2020). By incorporating Pml monooxygenase into the metabolic pathway, we have developed a completely biosynthetic approach to access 2,5-DMP-N-OX and 2,5-DMP-di-OX compounds. This work lays the foundation for new pyrazine synthesis platforms, ensuring compatibility between the production host and modifying enzymes in engineered metabolic pathways. For example, since L-threonine usually is the key building block, high L-threonine producers such as *Corynebacterium glutamicum* strains could be seen as hosts for a new type of metabolic engineering regarding pyrazines (Zhao et al., 2024), or naturally high pyrazine producers such as *Bacillus subtilis* (Kłosowski et al., 2021) and *Paenibacillus aceti* L14 (Li et al., 2017) could be utilized combining with modifying enzymes (e. g. Pml, XMO) to yield a range of oxyfunctionalized, high-valued pyrazine products. A key future target is the lipid-lowering drug acipimox, as our outlined metabolic pathways could facilitate its biosynthesis (Fig. 6). One strategy involves using XMO-produced MPCA as a substrate for Pml, which oxidizes it to acipimox. Alternatively, 2,5-DMP-N-OX produced by Pml could serve as a substrate for XMO, leading to the formation of the desired product. However, as discussed in our results, specific challenges related to Pml and XMO must be addressed to fully optimize these pathways.

Despite the promising potential for novel pyrazine biosynthetic pathways, overcoming bottlenecks is essential to achieve product titers comparable to those in *E. coli*. For instance, the highest recorded titer of 2,5-DMP, achieved through fermentative production using glucose as the carbon source, is 3.1 g L^−1^ (Zeng et al., 2024). Based on *E. coli* studies, three major steps for enhancing biosynthesis have emerged: (i) increasing the production of key intermediates (L-threonine, 2,5-DMP) via gene deletions targeting regulatory elements, transmembrane transport, and overexpression of genes involved in precursor supply (ii) gaining control over gene expression and protein biosynthesis (iii) and by balancing cofactor availability/depletion through the introduction of appropriate cofactor regeneration systems. Since not all of the planned precursor sink reactions (including the putative ones) have been addressed, they should be the primary target for increasing the availability of metabolic precursors, followed by the deletions targeting regulation and transport. Also, the availability of cofactors could be the possible hurdle in the presented metabolic pathway, as the oxidation of 2,5-DMP, along with the dehydrogenation of L-4-aspartyl phosphate and L-aspartate-4-semialdehyde, requires NAD(P)H (Fig. 1). Since only the Tdh-catalyzed conversion regenerates NADH, this limitation likely contributes to incomplete 2,5-DMP conversion as both XMO and Pml are capable of transforming significantly higher amounts of pyrazines under optimum conditions (Kiener, 1992; Petkevičius et al., 2021). Additionally, both previous studies and our findings have demonstrated that product yields depend significantly on the plasmid configuration and the induction parameters used (Gu et al., 2020). Gene expression from plasmids can be influenced by regulatory elements, plasmid size, plasmid copy number, and the metabolic state of the bacterial cell. The latest solution to this issue involves replacing protein biosynthesis from plasmids with protein biosynthesis from genomic integrons using CRISPR-Cas9 gene editing technology (Zeng et al., 2024). Equivalent approaches can also be applied to enhance pyrazine biosynthesis in *Pseudomonas*.

## 4 Conclusions

The use of L-threonine as a natural feedstock presents an efficient solution developing new synthesis pathways for high-value pyrazines. Our approach focused on minimizing complexity in metabolic engineering for 2,5-dimethylpyrazine (2,5-DMP) derivatives while considering differences between *P. putida* KT2440 and previous studies utilizing *E. coli* as a chassis. Through the identification of metabolic branches diverging from the L-threonine pathway and key biosynthesis genes, we directed carbon flux towards key intermediate 2,5-DMP synthesis while eliminating unwanted pathways through markerless deletions, resulting in the generation of strain KT2440Δ6. This strain exhibited superior retention of L-threonine compared to the wild-type *P. putida* KT2440 and was chosen for metabolic engineering. Heterologous overexpression of Tdh from *E. coli* DH5α enabled the connection between the L-threonine pathway and pyrazine skeleton synthesis, leading to successful 2,5-DMP production. Functionalization pathways leading to *N*-oxide and carboxylic acid products were explored by introducing monooxygenases PmlABCDEF and XylMABC, respectively. The engineered strains demonstrated the production of desired *N*-oxides and carboxylic acid derivatives of 2,5-DMP, highlighting the versatility and novelty of the designed biosynthesis pathway. Future research could focus on further refining the metabolic pathways, integrating advanced techniques such as CRISPR-based genome editing to enhance the productivity and efficiency of genetic modifications. Additionally, expanding the range of hosts capable of producing pyrazine derivatives, and combining them with various modifying enzymes, could extend the scope and availability of these pyrazine bio-products.

## 5 Materials and methods

### 5.1 Materials

2,5-Dimethylpyrazine, D-glucose, indole, metal salts, methanol, gentamicin, and TLC plates (TLC Silica gel 60 F254) were purchased from Sigma-Aldrich, Germany. Sucrose was obtained from Chempur, Poland. Agar and tryptone were products from Formedium, UK. Acrylamide, ampicillin, calcium chloride, potassium hydrophosphate, kanamycin, and sodium chloride were bought from Roth, Germany. Protein molecular weight marker, GeneJET™ Gel Extraction Kit, GeneJET™ PCR Purification Kit, Gene Ruler™ DNA Ladder Mix, and GeneRuler 1 kb DNA Ladder were ordered from Thermo Fisher Scientific, Lithuania. Ethidium bromide was purchased from SERVA, Germany. Chloroform was ordered from Honeywell Riedel-de Haen, Germany. Ethanol was a product from Vilniaus degtinė, Lithuania. Glycerol was bought from Barta a Cihlar, Czech Republic. Potassium dihydrogen phosphate, yeast extract, sodium hydroxide, tris(hydroxymethyl)aminomethane (Tris) were from Merck, Germany. L-arabinose was acquired from the Institute of Chemistry, Slovakia. L-threonine was received from Calbiochem Behring Corp, USA. Oligonucleotide DNA primers were ordered from Metabion, Germany. Sodium salicylate was obtained from Fisher Scientific, Lithuania.

### 5.2 Gene amplification and cloning

DNA fragments utilized for cloning were amplified via PCR employing 2X PhusionTM Plus PCR Master Mix (Thermo Fisher Scientific, Lithuania). Reaction samples were prepared following the manufacturer's guidelines. The PCR programs used were: i) program used for amplifying genes of interest: 98 °C 30 s; 98 °C 10 s, 60 °C 10 s, 72 °C 30 s/kb – 30 cycles; 72 °C for 7 min; ii) fusion PCR was used to obtain amplicons for the introduction of genetic mutations and the *thrA*^*S345F*^ gene using a gradient program: 98 °C for 30 s; 98 °C 10 s, 45–70 °C 10 s, 72 °C 30 s/kb - 5 cycles without primers in the reaction mixture, then primers were added and run for 30 cycles; 72 °C for 7 min 2X DreamTaq PCR Master Mix (Thermo Fisher Scientific, Lithuania) was employed for the selection of transformants. Reaction mixtures were prepared following the manufacturer's instructions. The PCR program utilized was as follows: 95 °C for 30 s; 95 °C for 10 s, 50-65 °C for 10 s, 72 °C for 1 min/kb – 30 cycles; followed by 72 °C for 7 min.

*P. putida* KT2440 biomass suspension was used as a matrix to obtain *ltaE* (PP_0321), *asnB* (PP_1750)*, dapA-II* (PP_2639)*, ilvA-I* (PP_3446), *ilvA-II* (PP_5149)*,* and *metX* (PP_5097) genes flanking regions used in markerless deletion experiments. Genes of XMO monooxygenase (GeneBank identifier D63341.1) were amplified directly from *Pseudomonas putida* mt-2 PaW1 (DSM 3931, Germany) biomass suspension. *Escherichia coli* DH5α biomass suspensions was used as a matrix to amplify threonine dehydrogenase *tdh* (GeneBank identifier EFH7827332.1) and *thrA* (GeneBank identifier WP_072662123.1). Genes of PmlABCDEF monooxygenase (GeneBank identifier MK037457.1) were cloned from pBAD2_Pml (Petkevičius et al., 2021). The PCR amplicons containing genes of interest were hydrolyzed with appropriate restriction enzymes (restriction sites introduced via PCR, a particular enzyme used is indicated by the name of primer, Table S2) and ligated to the properly digested protein expression vectors (pBAD2-MCS-1, pBNT, and pJNN). Alternatively, plasmid constructs were assembled by excising the target gene from one plasmid and transferring it to another backbone that had been cut with the same restriction enzymes. Recombinant genes were cloned containing ribosome binding sites (RBS) introduced via PCR using forward primer. Standard methods and techniques were employed for DNA manipulations (plasmid transformation, screening, isolation). Plasmid constructs and mutant deletions were confirmed by Sanger sequencing (Azenta Life Sciences, Germany). Plasmids containing multiple inserts were verified using Nanopore sequencing (SeqVision, Lithuania).

### 5.3 Markerless gene deletions in *Pseudomonas putida* KT2440

*P. putida* KT2440 mutant strains bearing multiple markerless gene disruptions were obtained by double crossover recombination technique according to the modified protocol by Oh et al. (2015). Two ∼500 bp regions upstream and downstream of the gene of interest were amplified by PCR (Table S1) directly from *P. putida* KT2440 biomass suspension. A kanamycin resistance cassette was amplified from FRT-PGK-gb2-neo-FRT PCR-template (Gene Bridges, Germany). Two genomic DNA fragments and a kanamycin resistance cassette were joined by overlap PCR to yield a DNA construct in a following sequence: upstream region of a gene targeted for deletion, downstream region of a gene targeted for deletion, and kanamycin resistance cassette. Next, this product was cloned into *Sma*I-digested pUC19_sacB suicide plasmid (Krasauskas et al., 2019). *P. putida* cells were transformed by electroporation (Choi et al., 2006). Kanamycin-resistant transformants were selected on LB agar plates supplemented with kanamycin (40 μg ml^−1^), grown at 30 °C overnight. The first crossover was confirmed by streaking colonies (20–30 of selected clones) on an LB agar plate containing 20 % sucrose, grown at 30 °C overnight. The positive clones demonstrated characteristic blurred shape and slower growth. A single sucrose-sensible clone was grown for 6 h in LB media at 37 °C and serial dilutions were plated on LB agar plates containing 20 % sucrose, and incubated overnight at 37 °C. This procedure aimed to isolate colonies that had undergone a secondary recombination event, leading to the loss of the plasmid integron from the genome. Hence, either a wild-type strain could be regenerated or a mutant lacking the target gene could be acquired. The identification of the mutant strain was achieved through colony PCR (Fig. S1) using primers flanking the appropriate gene.

### 5.4 Cultivation conditions

The bacterial strains used in this study are listed in Table 1. *E. coli* DH5a (Thermo Fischer Scientific, Vilnius, Lithuania) was used as a host for gene cloning and plasmid isolation. *Pseudomonas putida* KT2440 and its mutants were employed as hosts for recombinant protein production. The composition of growth media was as follows: LB – tryptone 10.0 g L^−1^, yeast extract 5.0 g L^−1^, NaCl 5.0 g L^−1^; MSM (slightly modified protocol by Hartmans et al., 1989) – K_2_HPO_4_ 1.55 g L^−1^, NaH_2_PO_4_ 0.85 g L^−1^, (NH_4_)_2_SO_4_ · 2H_2_O 2.0 g L^−1^, MgCl_2_ · 6H_2_O 0.1 g L^−1^, glucose 5.0 g L^−1^ (added after sterilization from 40 % stock solution), 1 mL of 1000x salt solution (10 mg L^−1^ EDTA, 1 mg L^−1^ ZnSO_4_ · 7H_2_O, 1 mg L^−1^ CaCl_2_ · 2H_2_O, 5 mg L^−1^ FeSO_4_ · 7H_2_O, 0.2 mg L^−1^ Na_2_MoO_4_ · 2H_2_O, 0.2 mg L^−1^ CuSO_4_, 0.4 mg L^−1^ CoCl_2_ · 6H_2_O, 1 mg L^−1^ MnCl_2_ · 2H_2_O as final concentrations). In the case of KT2440Δ6 cultivation, MSM medium was supplemented with 50x amino acid solution (lysine 0.4 g L^−1^, methionine 0.2 g L^−1^, isoleucine 0.2 g L^−1^, and asparagine 0.2 g L^−1^) as final concentrations). Before sterilization at 1 atm for 30 min pH adjustment to 7.0 was made. Potassium phosphate buffer, stock solution (27.2 g L^−1^ of KH_2_PO_4_, pH adjusted with KOH to 7.0 before sterilization) *E. coli* were grown at 30–37 °C temperature, while *Pseudomonas* strains were cultivated at 30 °C.

**Table 1:** Plasmids and strains used in this study.

Plasmid	Relevant characteristics	Source or reference
pUC19_sacB	Used as suicide vector for deletions in *Pseudomonas putida* KT2440	Krasauskas et al. (2019)
pBAD2-MCS-1	The amplicon containing regulatory elements of pBAD24 fused into the backbone of pBBR1MCS	Petkevičius et al. (2018)
pBNT	pBBR1 for replication in *E. coli* and *Pseudomonas*;	TU Delft, Netherlands (gift from Prof. Dirk Tischler)
	kanamycin resistance-cassette, salicylate-inducible	
	*nagR/pNagAa* promoter	
pJNN	ori RO1600 for *Pseudomonas* and ori ColE1 for *E.coli*; gentamicin resistance-cassette, salicylate-inducible *nagR/pNagAa* promoter	
FRT-PGK-gb2-neo-FRT	Template plasmid DNA for PCR amplification of the kanamycin cassette	Gene Bridges, Germany
pBAD2_Pml	Recombinant pBAD2-MCS-1 containing *pmlABCDEF* gene	Petkevičius et al. (2021)
pBNT_Pml	Recombinant pBNT containing *pmlABCDEF* gene	This study
pBAD2_tdh	Recombinant pBAD2-MCS-1 containing *tdh* gene from *E. coli* DH5α	This study
pBAD2_thrC	Recombinant pBAD2-MCS-1 containing *thrC* gene	This study
pJNN_thrC	Recombinant pJNN containing *thrC* gene	This study
pJNN_lysC	Recombinant pJNN containing *lysC* gene	This study
pJNN_thrA^S345F^	Recombinant pJNN containing *thrA* gene from *E. coli* DH5α with site-directed mutation of S345F	This study
pBNT_thrA^S345F^	Recombinant pBNT containing *thrA* gene from *E. coli* DH5α with site-directed mutation of S345F	This study
pBNT_lysC	Recombinant pBNT containing *lysC* gene	This study
pBNT_hom	Recombinant pBNT containing hom gene	This study
pBNT_thrC	Recombinant pBNT containing thrC gene	This study
pJNN_tdh_thrA^S345F^	Recombinant pJNN containing *thrA*^S345F^ and *tdh* genes	This study
pJNN_ thrB_thrC_thrA^S345F^	Recombinant pJNN containing *thrA*^S345F^, *thrB*, and *thrC* genes	This study
pBNT_thrB_thrC	Recombinant pBNT containing *thrB* and *thrC* genes	This study
pBAD2_XylMABC	Recombinant pBAD2-MCS-1 containing *xylAMBC* gene	This study
pBNT_XylMABC	Recombinant pBNT containing *XylAMBC* genes	This study

pUC19_sacB_asnB-del	Recombinant pUC19_sacB containing an assembled PCR construct for the deletion of the *asnB* gene in the *Pseudomonas putida* KT2440	This study

pUC19_sacB_dapaII-del	Recombinant pUC19_sacB containing an assembled PCR construct for the deletion of the *dapaII* gene in the *Pseudomonas putida* KT2440	This study

pUC19_sacB_ilvaI-del	Recombinant pUC19_sacB containing an assembled PCR construct for the deletion of the *ilvaI* gene in the *Pseudomonas putida* KT2440	This study

pUC19_sacB_ilvaII-del	Recombinant pUC19_sacB containing an assembled PCR construct for the deletion of the *ilvaII* gene in the *Pseudomonas putida* KT2440	This study

pUC19_sacB_ltaE-del	Recombinant pUC19_sacB containing an assembled PCR construct for the deletion of the *ItaE* gene in the *Pseudomonas putida* KT2440	This study

pUC19_sacB_metX-del	Recombinant pUC19_sacB containing an assembled PCR construct for the deletion of the *metX* gene in the *Pseudomonas putida* KT2440	This study

Strain		
*E. coli* DH5α	F^−^*endA1 glnV44 thi-1 recA1 relA1 gyrA96 deoR nupG purB20* φ80d*lacZ*ΔM15 Δ(*lacZYA-argF*)U169, hsdR17(*r*_*K*_^–^*m*_*K*_^+^), λ^–^	Thermo Fischer Scientific, Lithuania
*Pseudomonas putida* KT2440	Plasmid-free derivative of a toluene-degrading bacterium strain *Pseudomonas putida* mt-2	DSM 6125 DSMZ, Germany
KT2440Δ1	Derivative of *P. putida* KT2440 strain featuring markerless deletion of *ltaE* gene	This study
KT2440Δ2	Derivative of *P. putida* KT2440 strain featuring markerless deletions of *ltaE* and *asnB* genes	This study
KT2440Δ3	Derivative of *P. putida* KT2440 strain featuring markerless deletions of *ltaE*, *asnB,* and *dapA-II* genes	This study
KT2440Δ4	Derivative of *P. putida* KT2440 strain featuring markerless deletions of *ltaE*, *asnB, dapA-II,* and *ilvA-I* genes	This study
KT2440Δ5	Derivative of *P. putida* KT2440 strain featuring markerless deletions of *ltaE*, *asnB, dapA-II, ilvA-I* and *ilvA-II* genes	This study
KT2440Δ6	Derivative of *P. putida* KT2440 strain featuring markerless deletions of *ltaE*, *asnB, dapA-II, ilvA-I*, *ilvA-II,* and *metX* genes	This study

### 5.5 Induction conditions of recombinant plasmids and product biosynthesis

Recombinant plasmids were introduced into *P. putida* KT2440 strains via electroporation (Choi et al., 2006). The colonies obtained from the transformation were transplanted into 5 mL of liquid MSM medium (in 50 mL screw-capped plastic tubes) supplemented with the corresponding antibiotic (kanamycin 40 μg ml^−1^ or gentamicin 20 μg ml^−1^). The cultures were then incubated overnight at a thermoshaker (30 °C,180 rpm). The following day, the overnight culture was inoculated into 20 mL of MSM medium (100 mL Erlenmeyer glass flasks with screw caps) with the appropriate antibiotic at a ratio of 1:100. Cells were cultivated (30 °C,180 rpm) until the optical density of the suspension reached 0.8–1.0 (OD_600_). Subsequently, the appropriate inducer, either sodium salicylate (final concentration 1 mM) or arabinose (final concentration 0.2 %), was introduced into the flask to initiate the expression of the target genes. The cells were then incubated for approximately up to 72 h at 30 °C with agitation at 180 rpm, taking samples at fixed periods for HPLC-MS analysis.

L-threonine consumption as well as Tdh-catalyzed 2,5-DMP synthesis were executed in flasks containing 20 mL of potassium phosphate buffer (pH = 7.0, 20 mM), supplemented with L-threonine (two samples of 1.19 g L^−1^ and 2.38 g L^−1^) and 1.5 g L^−1^ CDW of appropriate cell biomass. The incubation was carried out for 72 h at 30 °C with agitation at 180 rpm, taking samples at fixed periods for HPLC-MS analysis.

### 5.6 HPLC-MS analysis of the biosynthesis products

The 0.5 mL sample from the cultivation broth was transferred to a 1.5 mL tube and mixed with an equal volume of acetonitrile. After centrifugation at 12,000 *g* for 5 min, the supernatant (0.5 mL) was analyzed by injection into a high-performance liquid chromatography (HPLC) system. HPLC-MS analysis was conducted using a Shimadzu high-performance liquid chromatography system from Japan, equipped with a photo diode array (PDA) detector and a mass spectrometer (LCMS-2020; Shimadzu) with an ESI source. Chromatographic separation was conducted utilizing a YMC-Pack Pro C18, 3 × 150 mm column (YMC, Kyoto, Japan) at 40 °C, employing a mobile phase composed of 0.1 % aqueous formic acid (solvent A) and acetonitrile (solvent B) in a gradient elution mode ranging from 5 % to 95 % B. Mass spectra were recorded from m/z 50 to m/z 700 at 350 °C, with a 250 °C DL temperature and a ±4500V interface voltage (neutral DL/Qarray voltage), using N_2_ as the nebulizer and drying gas. Data analysis was performed using LabSolutions LCMS software.

The presence of L-threonine was determined by identifying a specific signal (*m/z* [M+H]^+^ = 120) in the mass spectrometry chromatogram, using L-threonine standard solutions ranging from 0.1 to 10 mM. The concentrations of 2,5-DMP, 2,5-DMP-N-oxide, 2,5-DMP-1,4-dioxide, and MPCA were determined from standard calibration curves made by integrating the light absorption curves (277 nm, 260 nm, 300 nm, and 273 nm, respectively) of appropriate pyrazine products in the HPLC chromatogram, using standard solutions ranging from 0.05 to 5 mM. Assays were performed in triplicates (at least three independent biological repetitions), mean values were presented, and the standard deviation was less than 10 %. A product titre of the reaction was described as the total amount (mg) of product formed in a conversion mixture (L) during the cultivation.

### 5.7 Isolation of pyrazine products from the cultivation broth

The appropriate strain was grown in 20 mL of MSM medium with the appropriate antibiotic for up to 72 h at 30 °C with agitation at 180 rpm. In the synthesis process of pyrazine *N*-oxides, the reaction mixture was separated from the biomass through centrifugation at 8000*g* for 30 min. The supernatant's volume was then reduced under vacuum to 5–10 mL before transferring to a separation funnel. Subsequently, the aqueous phase underwent washing with 20–30 mL of chloroform, repeated at least three to four times. The organic phases were then combined and dried using anhydrous Na_2_SO_4_ and reduced under vacuum to 1–2 mL. The resulting mixture was transferred for the purification procedure by silica gel column chromatography with CHCl_3_-MeOH (5:1). Similarly, the reaction mixture was separated from the biomass through centrifugation and reduced under vacuum to 5–10 mL in the isolation of MCPA and 2,5-DMP-OH. Then, the pH of the solution was adjusted to acidic (pH 3–4) by the HCl solution before transferring it to a separation funnel. The aqueous phase was washed with 20–30 mL of ethyl acetate, and repeated at least three to four times. The organic phases were combined and dried using anhydrous Na_2_SO_4_ and reduced under vacuum to 1–2 mL. before transferring for purification by silica gel column chromatography with CHCl_3_-MeOH (5:1).

### 5.8 ^1^H and ^13^C NMR analysis

Purified compounds (2,5-DMP-N-oxide, 2,5-DMP-1,4-dioxide, 2,5-DMP-OH, and MPCA) were subjected to NMR analysis. The structure of 2,5-DMP was assigned by comparing HPLC-MS data with commercially available standards without further purification and analyses. NMR spectra were acquired using DMSO-d6 or CDCl_3_ solvents on an Ascend 400 spectrometer operating at 400 MHz for ^1^H NMR and 101 MHz for ^13^C NMR (Bruker, MA, USA). Chemical shifts (δ) are reported in ppm relative to the solvent resonance signal, serving as an internal standard.

2,5-dimethylpyrazine 1-oxide (2,5-DMP-N-oxide) obtained as a white solid. ^1^H BMR (400 MHz, DMSO-d_6_): δ = 2,28 (s, 3H, CH), 2,38 (s, 3H, CH), 8,30 (s, 1H, CH), 8,47 (s, 1H, CH). ^13^C BMR (101 MHz, DMSO-d_6_): δ = 13.98, 21.13, 132.34, 140.92, 147.10, 155.19.

2,5-dimethylpyrazine 1,4-dioxide (2,5-DMP-di-OX) was obtained as a yellowish solid. ^1^H BMR (400 MHz, DMSO-d6): δ = 2,23 (s, 6H, CH), 8,50 (s, 2H, CH). ^13^C BMR (101 MHz, DMSO-d6): δ = 14.57, 135.24, 144.41.

5-methyl-2-pyrazinecarboxylic acid (MPCA) was isolated as a pale grey solid. ^1^H BMR (400 MHz, DMSO-d6): δ = 2,59 (s, 3H, CH), 8.68 (d, *J* = 1.5 Hz, 1H, CH), 9.06 (d, *J* = 1.4 Hz, 1H, CH). ^13^C BMR (101 MHz, DMSO-d6): δ = 21.88, 141.46, 145.09, 144.75, 157.78, 165.75.

### 5.9 Writing and editing

The manuscript's language was improved through the use of ChatGPT 3.5 (OpenAI, 2023). The authors affirm that AI was solely employed as an editing tool for grammar and style, with the experimental data, presentation, writing, and analysis representing authentic work conducted by the authors.

## CRediT authorship contribution statement

**Vytautas Petkevičius:** Writing – review & editing, Writing – original draft, Data curation, Conceptualization. **Justė Juknevičiūtė:** Writing – review & editing, Data curation. **Domas Mašonis:** Writing – review & editing, Data curation. **Rolandas Meškys:** Writing – review & editing, Supervision, Conceptualization.

## Declaration of competing interest

The authors declare that they have no known competing financial interests or personal relationships that could have appeared to influence the work reported in this paper.

## Data Availability

Data will be made available on request.
