# Supplementary material for: Synthetic pathways for microbial biosynthesis of valuable pyrazine derivatives using genetically modified Pseudomonas putida KT2440
Source: Metab Eng Commun. 2025 Mar 30;20:e00258. doi: 10.1016/j.mec.2025.e00258 (PMC11999294; doi:10.1016/j.mec.2025.e00258)

**Synthetic Pathways for Microbial Biosynthesis of Valuable Pyrazine Derivatives Using Genetically Modified *Pseudomonas putida* KT2440**

**Supporting information**

Vytautas Petkevičius^1^, Justė Juknevičiūtė^1^, Domas Mašonis^1^, Rolandas Meškys^1^

*^1^ Department of Molecular Microbiology and Biotechnology, Institute of Biochemistry, Life Sciences Center, Vilnius University, Saulėtekio 7, Vilnius LT-10257, Lithuania*

*For correspondence. E-mail vytautas.petkevicius@bchi.vu.lt; Tel. +37067979857*

[Table S1. 2](#_Toc177037019)

[Table S2 3](#_Toc177037020)

[Gel analysis of markerless gene deletions 4](#_Toc177037021)

[SDS-PAGE analysis of individually expressed recombinant proteins in *P. putida* strains 5](#_Toc177037022)

[HPLC-MS chromatograms of bioconversions 9](#_Toc177037023)

[^1^H and ^13^C NMR spectra of pyrazine products 15](#_Toc177037024)

Table S1. Primers used in this study for gene deletion experiments.

| Primer name | Sequence (5‘→ 3‘) |
| --- | --- |
| asnB_gal_F | ATGGGCTTTCATCGGAAGTCCTTACCGGTTTTTCC |
| asnB_gal_R | TAGTGAGGGTTAATTACGAAATTTCCGTCGATCTG |
| asnB_prie_F | CGTCCTCTGCCAGCCAATAC |
| asnB_prie_R | AACCGGTAAGGACTTCCGATGAAAGCCCATGAAATC |
| dapa1_gal_F | GGCAGGAGCAGTTACGCATGAAGCGACTGGCTGGTC |
| dapa1_gal_R | TAGTGAGGGTTAATTCCAAGCGATGCCGACAGTTCA |
| dapa1_prie_F | GGGTCAGGCCGTGCTTCTTGC |
| dapa1_prie_R | CCAGTCGCTTCATGCGTAACTGCTCCTGCCGGAAA |
| dapa2_gal_F | ATCAGGAGTTTTTGCAACGGTTGATGGCGGGTTGAC |
| dapa2_gal_R | TAGTGAGGGTTAATTTTACCCAGGTGTCAGCACCCC |
| dapa2_prie_F | CCTAAGTGACCAGTGGGTACTG |
| dapa2_prie_R | CCGCCATCAACCGTTGCAAAAACTCCTGATGTTGAC |
| ilva1_gal_F | GCATTGGCCGCACAGGAGTCAGAGAGAAAAAACCC |
| ilva1_gal_R | TAGTGAGGGTTAATTGCGCAGTCGACCGTGCTGTC |
| ilva1_prie_F | TGGCGCTGATGATCTACTCGC |
| ilva1_prie_R | TTTTCTCTCTGACTCCTGTGCGGCCAATGCACGC |
| ilva2_gal_F | CCCTAGCTTAGCCGCCGGGAAAGCATCACTGGGCCG |
| ilva2_gal_R | TAGTGAGGGTTAATTCTTGCTGCCGTCGGCGATGCAG |
| ilva2_prie_F | GTAAACCGCAACCCTACC |
| ilva2_prie_R | AGTGATGCTTTCCCGGCGGCTAAGCTAGGGCTG |
| knm_F | AATTAACCCTCACTAAAGGG |
| knm_R | TAATACGACTCACTATAGGG |
| ltaE_gal_F | TCACAGGACCGTGCCTGAGCGAGAGCCGGGGCC |
| ltaE_gal_R | TAGTGAGGGTTAATTAGACCAACCTGCTCGCGTTG |
| ltaE_prie_F | gcttgctggtgtagttctccgaggc |
| ltaE_prie_R | CCCGGCTCTCGCTCAGGCACGGTCCTGTGAACGAC |
| metx_gal_F | GCGTGAGCAGGGTCGGGACAGCATGAGAGCCGATCTG |
| metx_gal_R | TAGTGAGGGTTAATTCGAGTACCTTGGCCCGGCGTT |
| metx_prie_F | GGCTGGATACCTCGTCGCTACTG |
| metx_prie_R | GCTCTCATGCTGTCCCGACCCTGCTCACGCTTGACGG |
| pvdh_gal_F | AACAGTGGCATGCAGGGTACAACCCGGGCAAGGCTG |
| pvdh_gal_R | TAGTGAGGGTTAATTCATGGCGGCGATGAAGCCGTG |
| pvdh_prie_F | GTCTGAGCGGCGATCACACT |
| pvdh_prie_R | TGCCCGGGTTGTACCCTGCATGCCACTGTTTGACGC |
| thrA_C1034T_F | GCGCCCGTATTTTCGTGGTGCTGATTAC |
| thrA_C1034T_R | ATCAGCACCACGAAAATACGGGCGCGTG |

Table S2**.** Primers used in this study for gene cloning experiments.

| Primer name | Sequence (5‘→ 3‘) |
| --- | --- |
| thrA_mut_Xba_F | ATTATCTAGAAATAATTTTGTTTAACTTTAAGAAGGAGATATACCATG CGAGTGTTGAAGTTCG |
| thrA_suliet_F | TTTAACTTTAAGAAGGAGATATACCATGCGAGRGTTGAAG |
| thrA_not_R | ATTAGCGGCCGCTCAGACTCCTAACTT |
| Tdh_ecoRI _F | TTAAGAATTCTTAATCCCAGCTCAG |
| Tdh_suliet_F | TTTAACTTTAAGAAGGAGATATACCATGAAAGCGTTATCC |
| Tdh_hind _R | TAATAAGCCTTTAATCCCAGCTCAG |
| thrB_ecoRI_F | ATATGAATTCTTTAAGAAGGAGATATACCATGTCAGTCTTCACCCCCGTGACC |
| thrB_suliet_R | GGTATATCTCCTTCTTAAAGTTAAACTACAGCGCGAACGG |
| thrC_suliet_F | TTTAACTTTAAGAAGGAGATATACCATGCGCTATATCAGT |
| thrC_xba_R | ATTATCTAGATTACAGCGGCTTGCCGCGGTTGCCGTGC |
| xylM_ecorI_F | ATTAGAATTCTTTAAGAAGGAGATATACCATGGACACGCTTCGT |
| xylA_knp_R | ATTAGGTACCCTAGCAAGGAGGTCTATTAT |
| xylB_knp_R | ATTAGGTACCTCAACCAATCCGGAGTACC |
| xylC_F | TAAGGAGGCATAATTATGCGGGAAAC |
| xylC_R | GACTCAAAATGGGTAATTAGCTGG |
| hom_nhe_R | TaatgctagcTTAGTTGAGCTGCTCGACGCG |
| hom_rbs_ecori_F | Tatagaattcacgtgtcggccgttc |
| lysC_rbs_ecori_F | Tatagaattcgccctgtatgggctg |
| lysC_xba_R | TACTTCTAGATTACTCGCCCTGTCGGGCAGG |
| thrB_not_R | ttatgcggccgcCTACAGCGCGAACGGCAGGTGCAATG |
| thrB_rbs_xba_F | Attatctagagaagtgcccgtatgg |
| thrC_ecor_F | ATTAGAATTCATGCGCTATATCAGTACCCGCGG |
| thrC_xho_R | ttaactcgagTTACAGCGGCTTGCCGCGGTTG |

# Gel analysis of markerless gene deletions


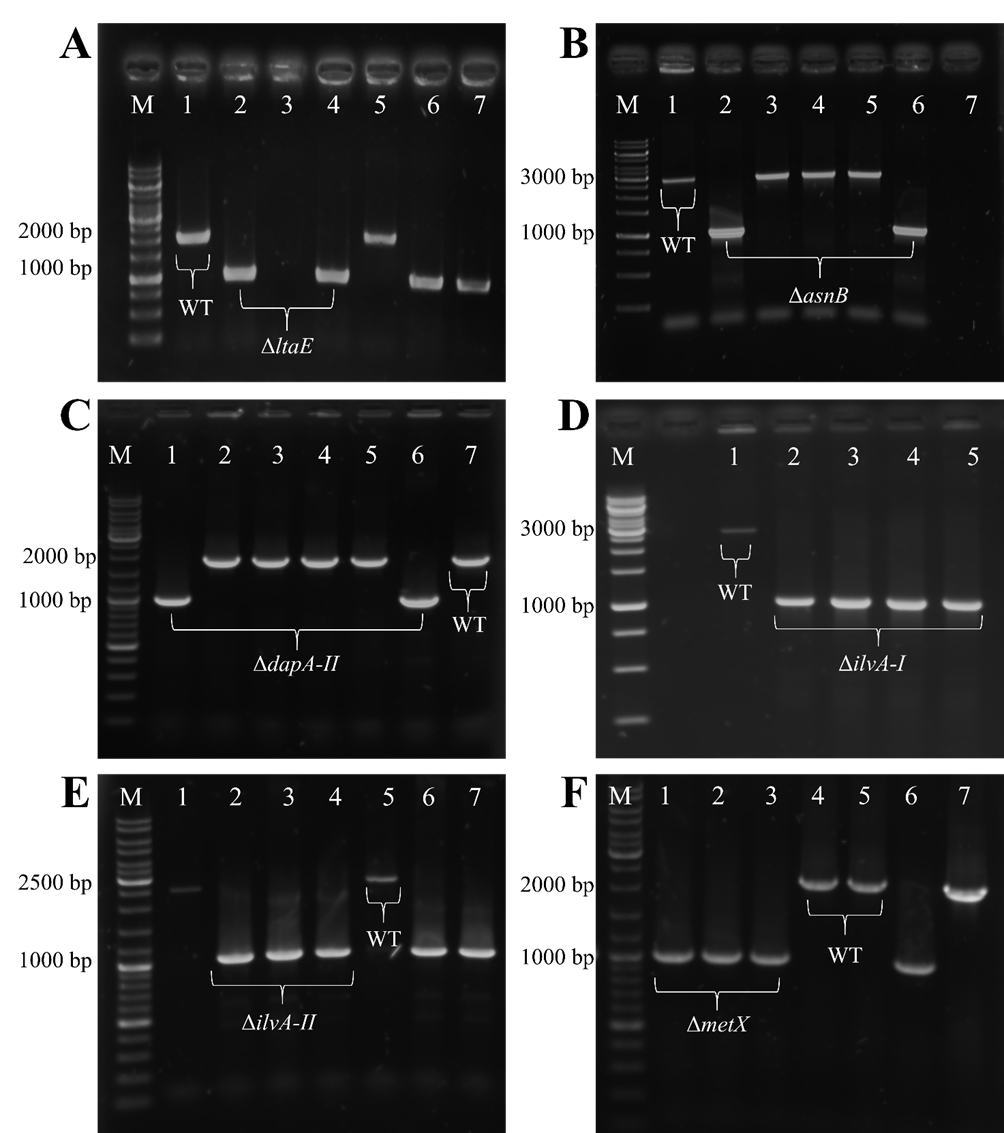


**Fig. S1**. Analysis of PCR products from genetically modified colonies of *Pseudomonas putida* KT2440 in agarose gel. Larger products indicate wild type (WT) phenotype, where smaller fragments show that the appropriate gene has been lost (successful deletion) in the flanking region. M - DNA size marker GeneRuler 1 kb DNA Ladder, 1-7 - PCR products of tested colonies. Analysis of each gene deletion is as follows: A – Δ*ltaE*, B – Δ*asnB*, C – Δ*dapA-II*, D – Δ*ilvA-I*, E – Δ*ilvA-II*, F – Δ*metX*.

# SDS-PAGE analysis of individually expressed recombinant proteins in *P. putida* strains


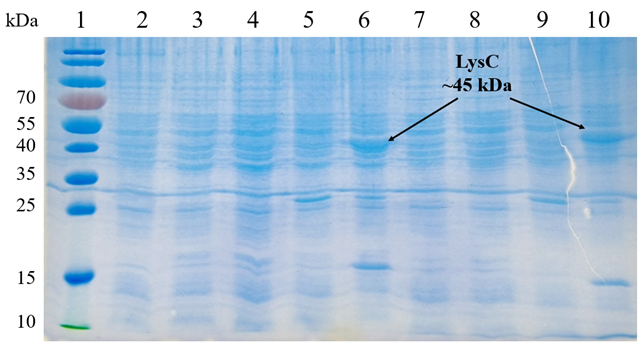


**Fig. S2** Protein analysis of plasmid-transformed *P. putida* KT2440 strains. Induction was performed at 30 °C for 16 h, first run. The gel lanes are as follows: Lane 1 – molecular weight marker; Lane 2 – negative control, supernatant fraction from KT2440 with pBNT; Lane 3 – supernatant fraction from KT2440Δ6 with thrB_pJNN; Lane 4 – supernatant fraction from KT2440Δ6 with thrC_pJNN_; Lane 5 – supernatant fraction from KT2440Δ6 with hom_pBNT plasmid; Lane 6 – supernatant fraction from KT2440Δ6 with lysC_pBNT; Lane 7 – total fraction from KT2440Δ6 with thrB_pJNN; Lane 8 – total fraction from KT2440Δ6 with thrC_pJNN; Lane 9 – total fraction from KT2440Δ6 with hom_pBNT; Lane 10 – total fraction from KT2440Δ6 with lysC_pBNT. The theoretical protein sizes for the LysC, Hom, ThrC, and ThrB proteins, calculated from their amino acid sequences, are 44.6 kDa, 46.2 kDa, 51.6 kDa, and 35.2 kDa, respectively.


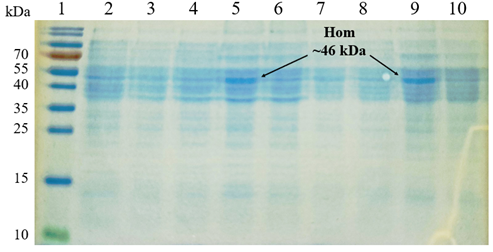


**Fig. S3** Protein analysis of plasmid-transformed *P. putida* KT2440 strains. Induction was performed at 30 °C for 16 h, second run. The gel lanes are as follows: Lane 1 – molecular weight marker; Lane 2 – negative control, supernatant fraction from KT2440 with pBNT; Lane 3 – supernatant fraction from KT2440Δ6 with lysC_pBNT; Lane 4 – supernatant fraction from KT2440Δ6 with thrB_pJNN_; Lane 5 – supernatant fraction from KT2440Δ6 with hom_pBNT plasmid; Lane 6 – supernatant fraction from KT2440 with thrC_pBNT; Lane 7 – total fraction from KT2440Δ6 with lysC_pJNN; Lane 8 – total fraction from KT2440Δ6 with thrB_pJNN; Lane 9 – total fraction from KT2440Δ6 with hom_pBNT; Lane 10 – total fraction from KT2440Δ6 with thrC_pBNT. The theoretical protein sizes for the LysC, Hom, ThrC, and ThrB proteins, calculated from their amino acid sequences, are 44.6 kDa, 46.2 kDa, 51.6 kDa, and 35.2 kDa, respectively.


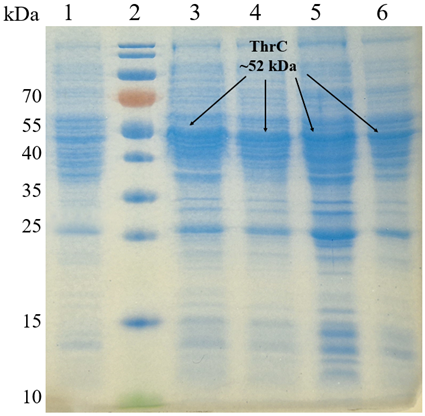


**Fig. S4** Protein analysis of *P. putida* KT2440 strains transformed with the pJNN_thrC or pBAD2_thrC plasmids. Induction was performed at 30 °C for 16 h. Lane 1 – negative control, supernatant fraction from KT2440 with pBAD2-MCS1 plasmid. Lane 2 – molecular weight marker. Lane 3 – total fraction from KT2440Δ6 with thrC_pBAD2 plasmid. Lane 4 – supernatant fraction from KT2440Δ6 with thrC_pBAD2 plasmid. Lane 5 – total fraction from KT2440Δ6 with thrC_pJNN plasmid. Lane 6 – supernatant fraction from KT2440Δ6 with thrC_pJNN plasmid. The theoretical molecular weight of the ThrC protein, calculated from the amino acid sequence, is 51.6 kDa.


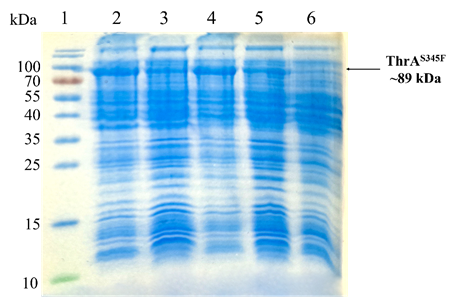


**Fig. S5** Protein analysis of *P. putida* Δ6 KT2440 strain transformed with the pBNT_thrA^S345F^ plasmid. Lane 1 – molecular weight marker. Lane 2 – total fraction, induction was carried out at 30 °C for 16 h. Lane 3 – total fraction, induction was carried out at 16 °C for 16 h. Lane 4 – supernatant fraction, induction was carried out at 30 °C for 16 h. Lane 5 – supernatant fraction, induction was carried out at 16 °C for 16 h. Lane 6 – negative control, supernatant fraction from KT2440 with pBNT plasmid.

# HPLC-MS chromatograms of bioconversions


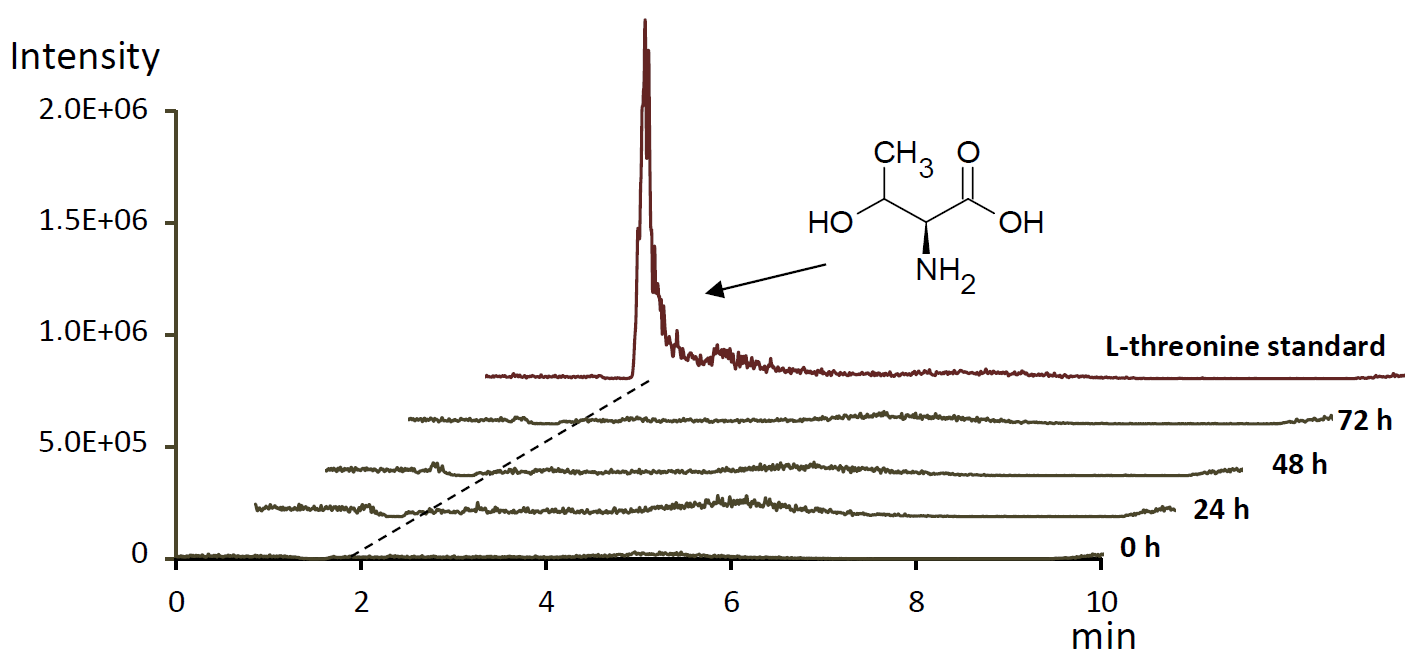


**Chromatogram S1.** A time-course cultivation of the *P. putida* KT2440 wild type strain in MSM medium. The chromatograms correspond to the extracted ion count of the L-threonine standard (120 m/z [M+H]+). The standard sample was used at a final concentration of 5 mM (shown in brown).


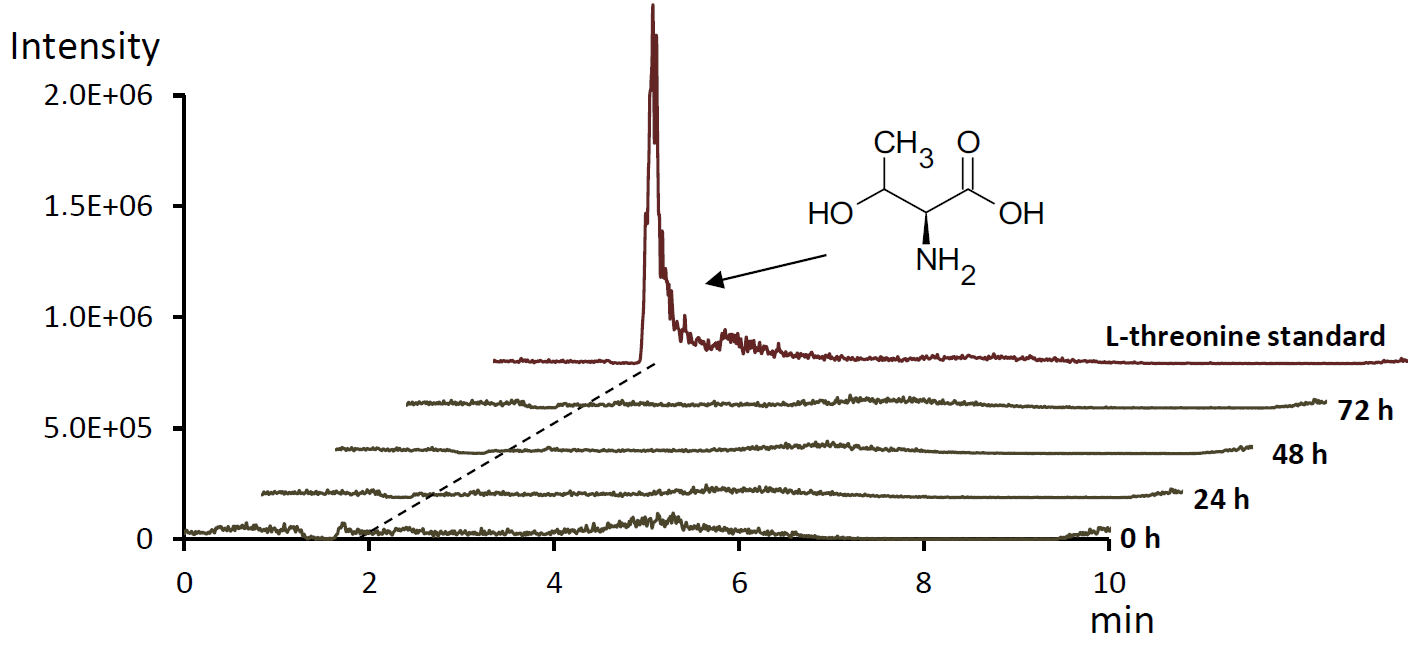


**Chromatogram S2.** A time-course cultivation of the *P. putida* KT2440Δ6 strain in MSM medium. The chromatograms correspond to the extracted ion count of the L-threonine standard (120 m/z [M+H]+). The standard sample was used at a final concentration of 5 mM (shown in brown).


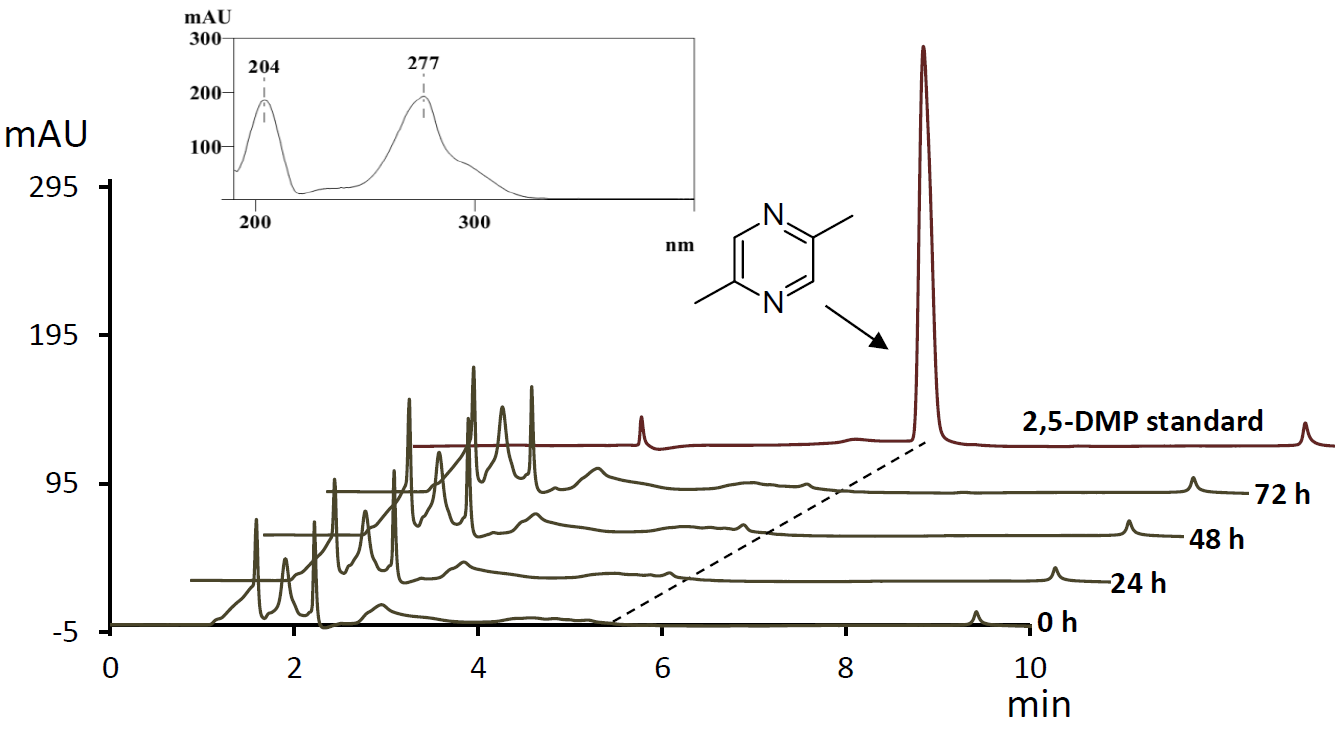


**Chromatogram S3.** A time-course cultivation of the *P. putida* KT2440 wild type strain in MSM medium containing the pBAD2_tdh plasmid, without the addition of L-threonine. The standard curve for 1 mM 2,5-DMP is shown in brown. The curves were measured at a wavelength of 277 nm.


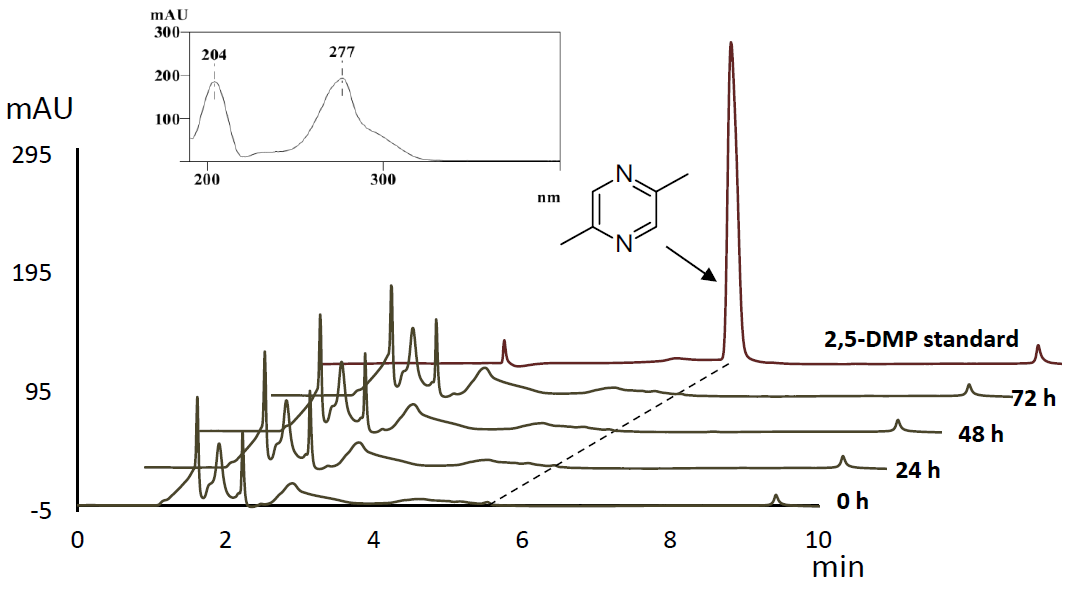


**Chromatogram S4.** A time-course cultivation of the *P. putida* KT2440Δ6 strain in MSM medium containing the pBAD2_tdh plasmid, without the addition of L-threonine. The standard curve for 1 mM 2,5-DMP is shown in brown. The curves were measured at a wavelength of 277 nm.


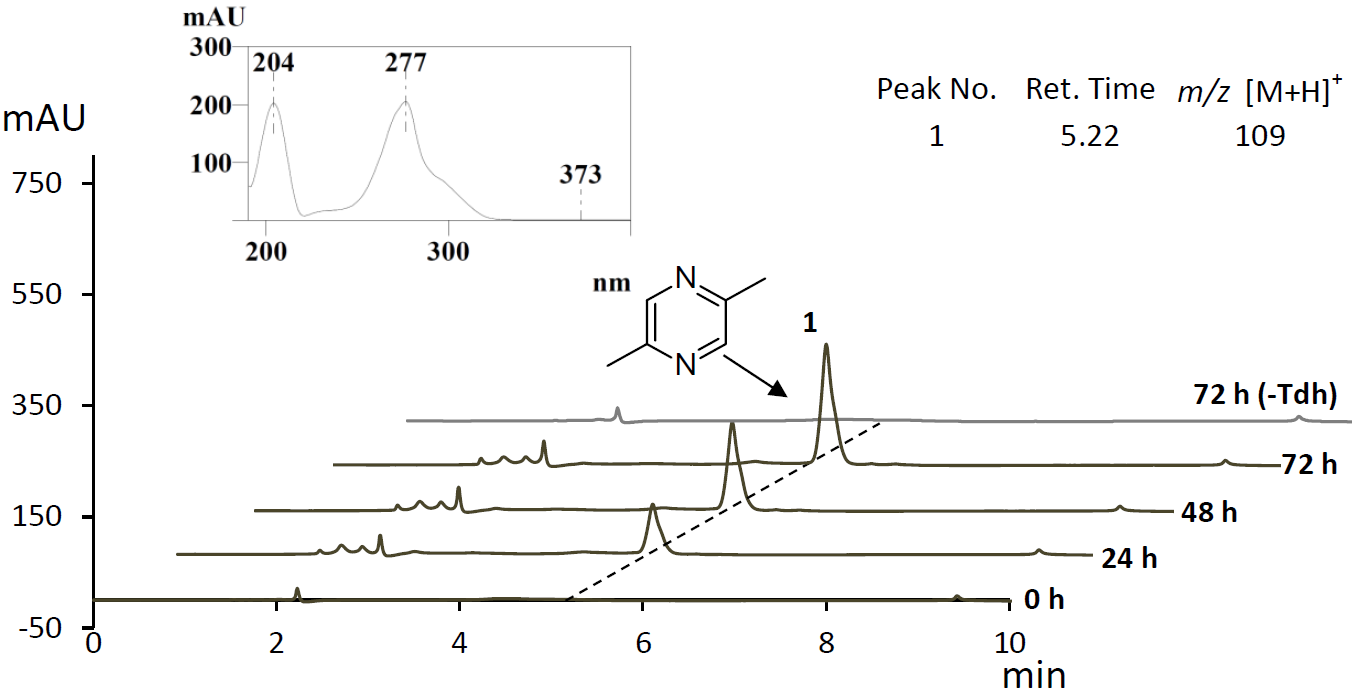


**Chromatogram S5.** A time-course of 2,5-DMP synthesis by *P. putida* KT2440 wild type strain harboring pBAD2_tdh plasmid and without a plasmid (-Tdh), supplemented with 10 mM of L-threonine (277 nm wavelength).


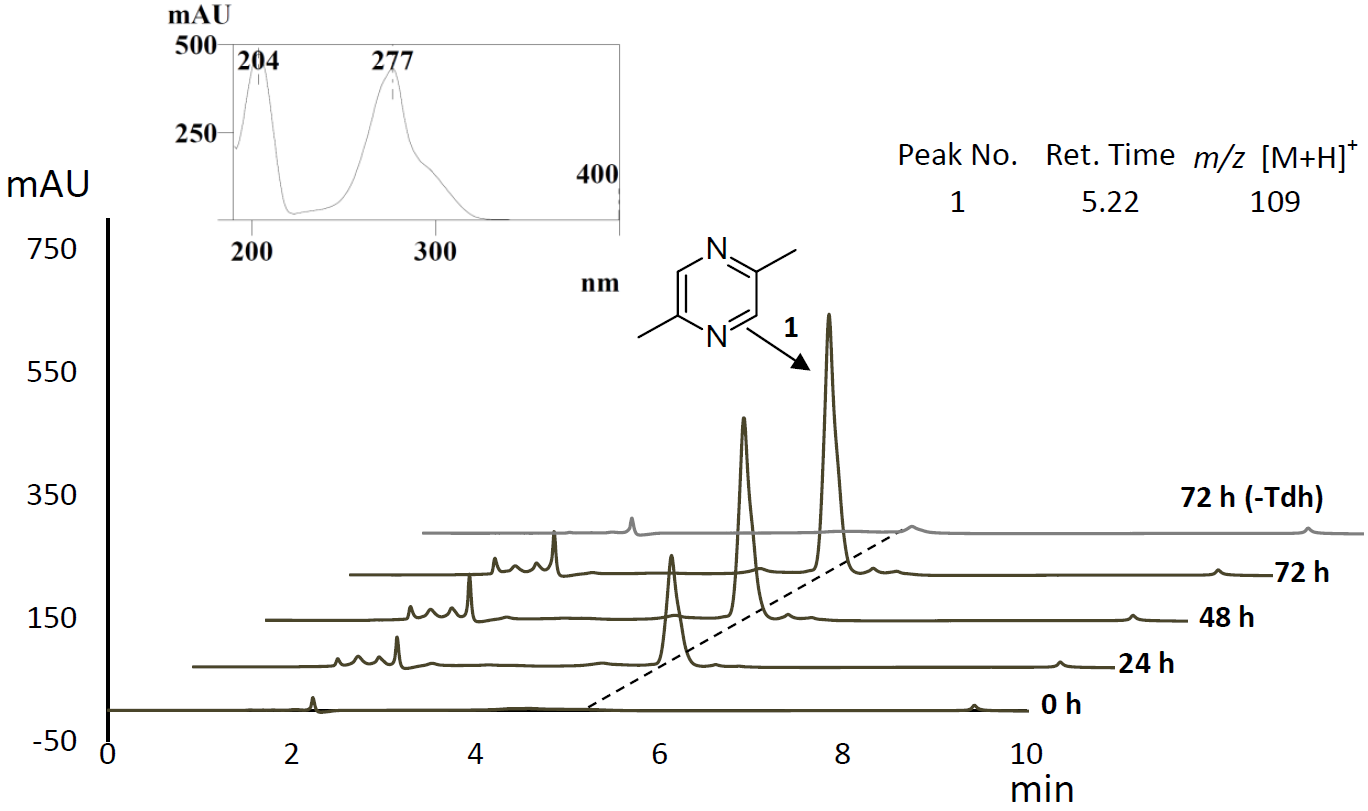


**Chromatogram S6.** A time-course of 2,5-DMP synthesis by *P. putida* KT2440 wild type strain harboring pBAD2_tdh plasmid and without a plasmid (-Tdh), supplemented with 20 mM of L-threonine (277 nm wavelength).


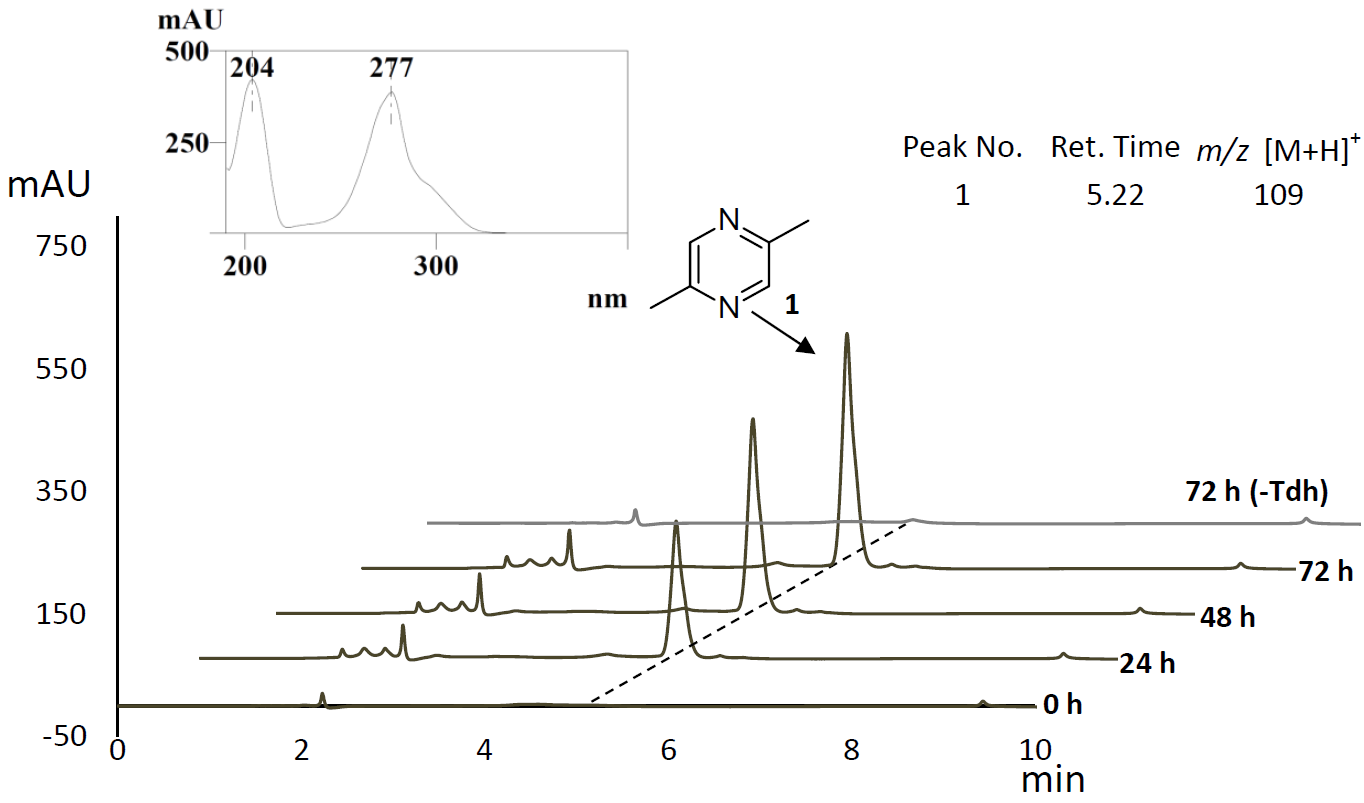


**Chromatogram S7.** A time-course of 2,5-DMP synthesis by *P. putida* KT2440Δ6 strain harboring pBAD2_tdh plasmid and without a plasmid (-Tdh), supplemented with 10 mM of L-threonine (277 nm wavelength).


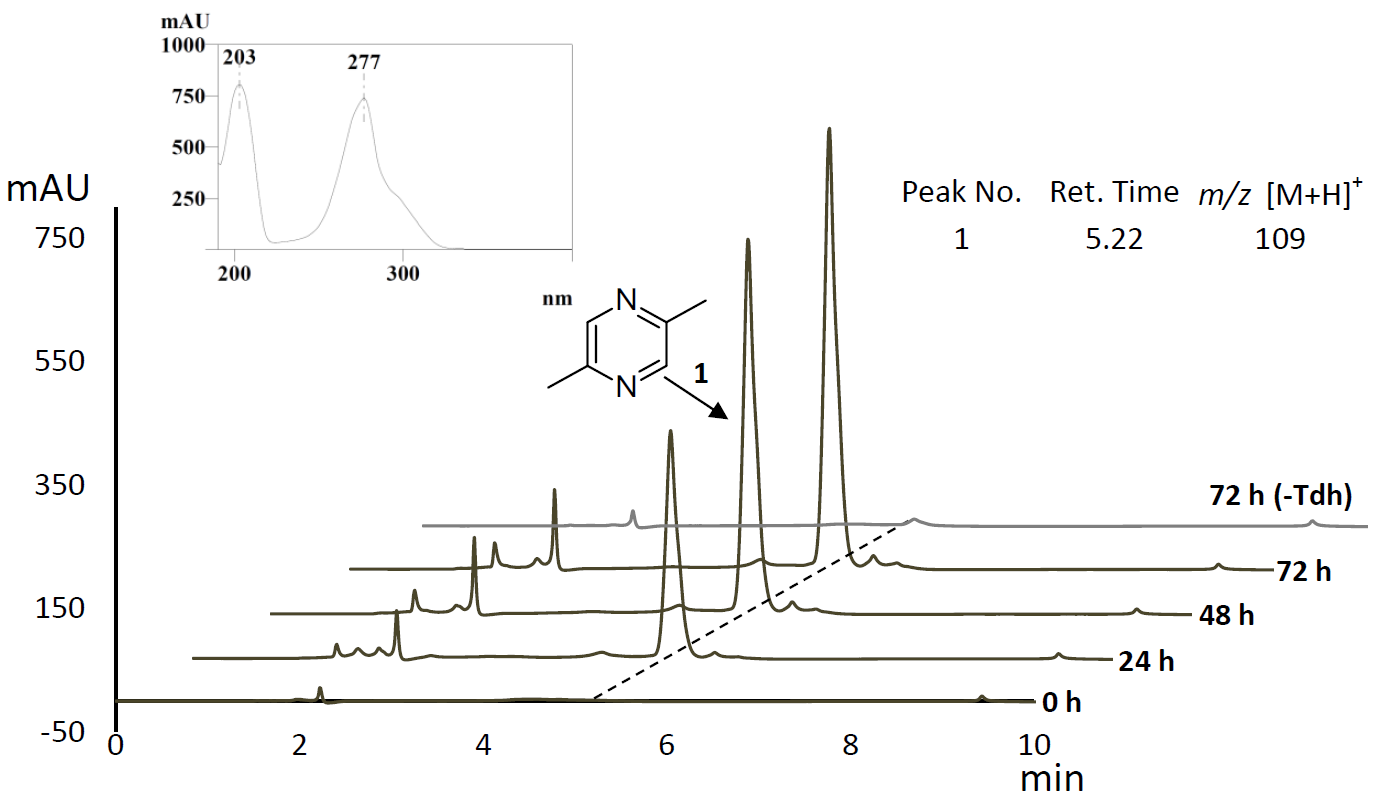


**Chromatogram S8.** A time-course of 2,5-DMP synthesis by *P. putida* KT2440Δ6 strain harboring pBAD2_tdh plasmid and without a plasmid (-Tdh), supplemented with 20 mM of L-threonine (277 nm wavelength).


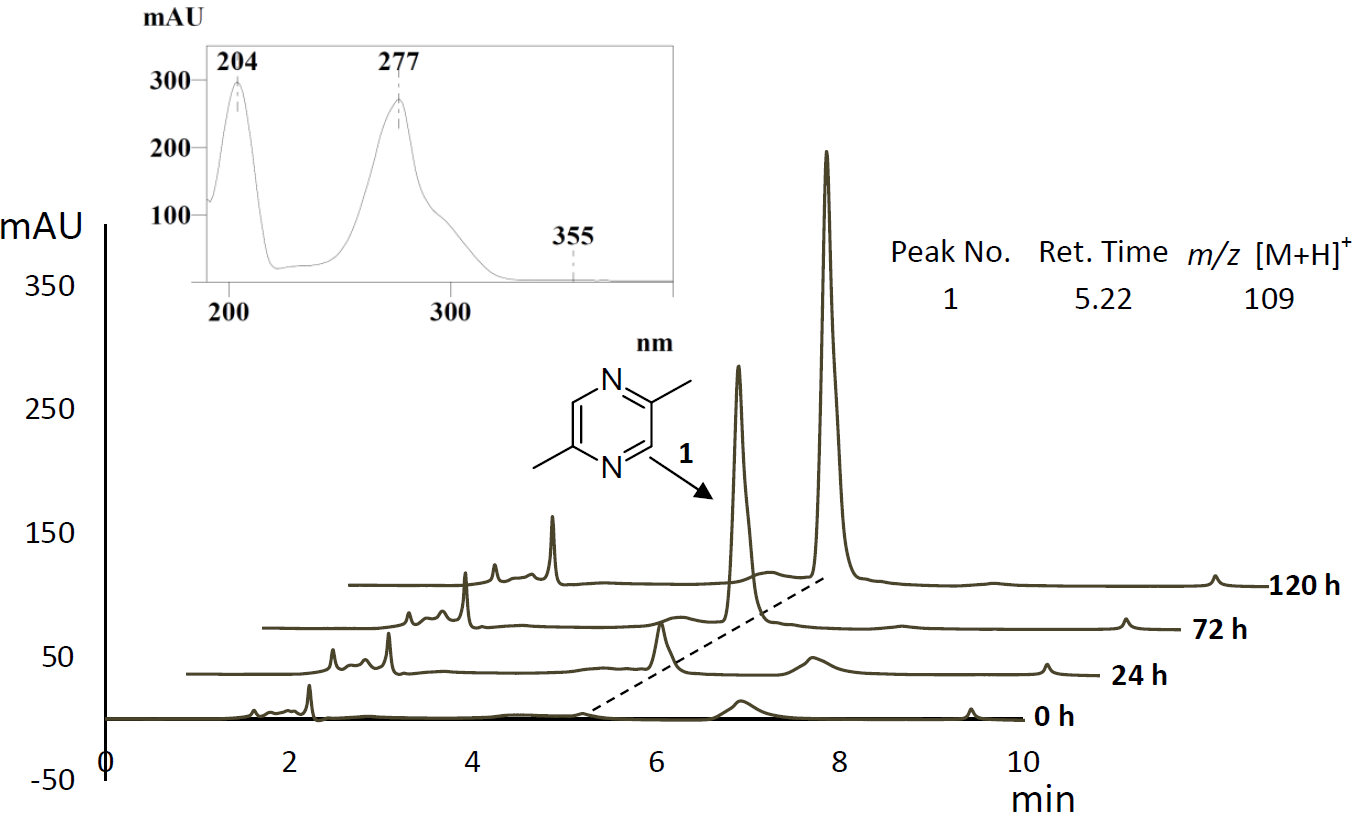


**Chromatogram S9.** A time-course of 2,5-DMP synthesis by *P. putida* KT2440Δ6 strain harboring pJNN_tdh_thrA^S345F^ plasmid, cultivated in MSM medium without the additional L-threonine (277 nm wavelength).


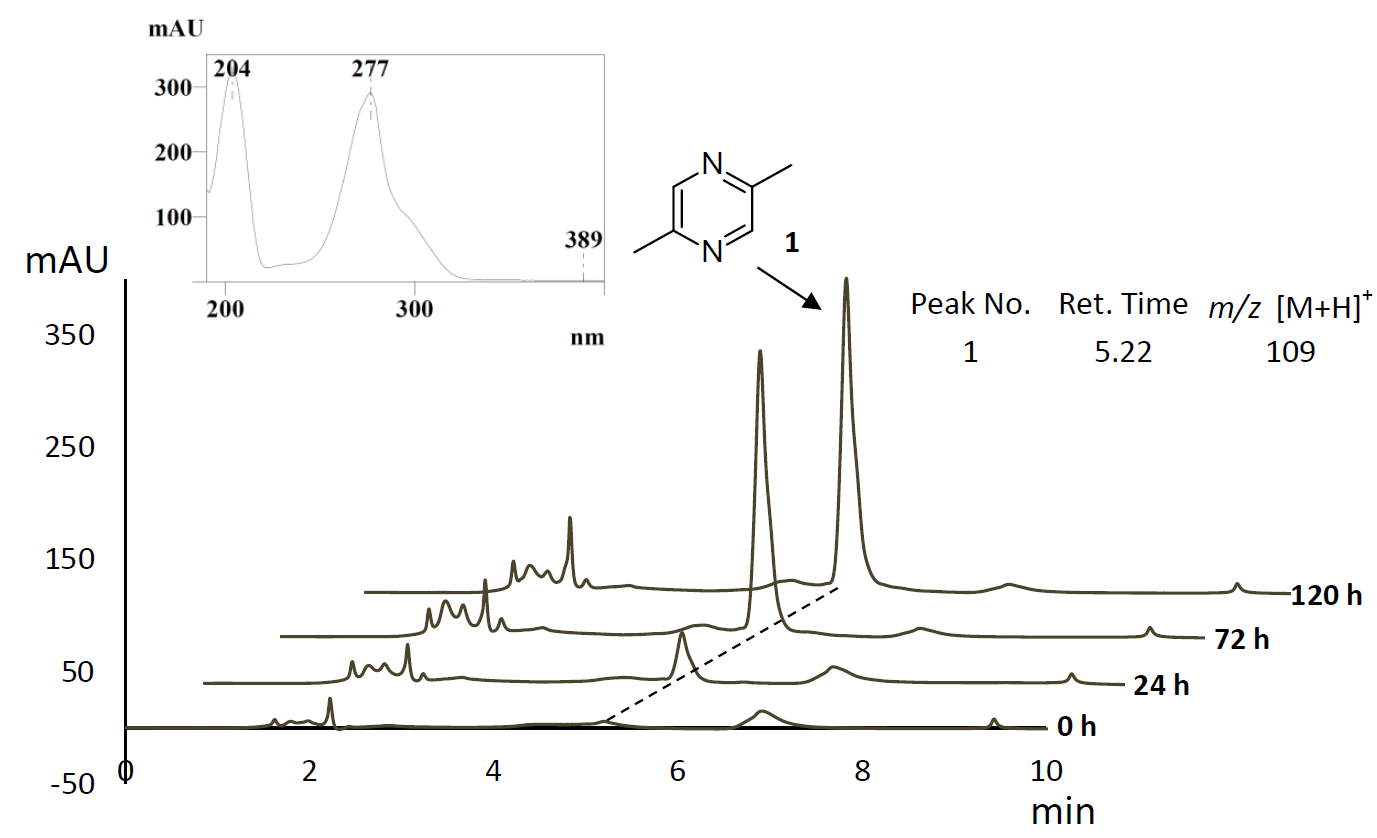


**Chromatogram S10.** A time-course of 2,5-DMP synthesis by *P. putida* KT2440Δ6 strain harboring pBAD2_tdh and pJNN_thrA^S345F^ plasmids, cultivated in MSM medium without the additional L-threonine (277 nm wavelength).


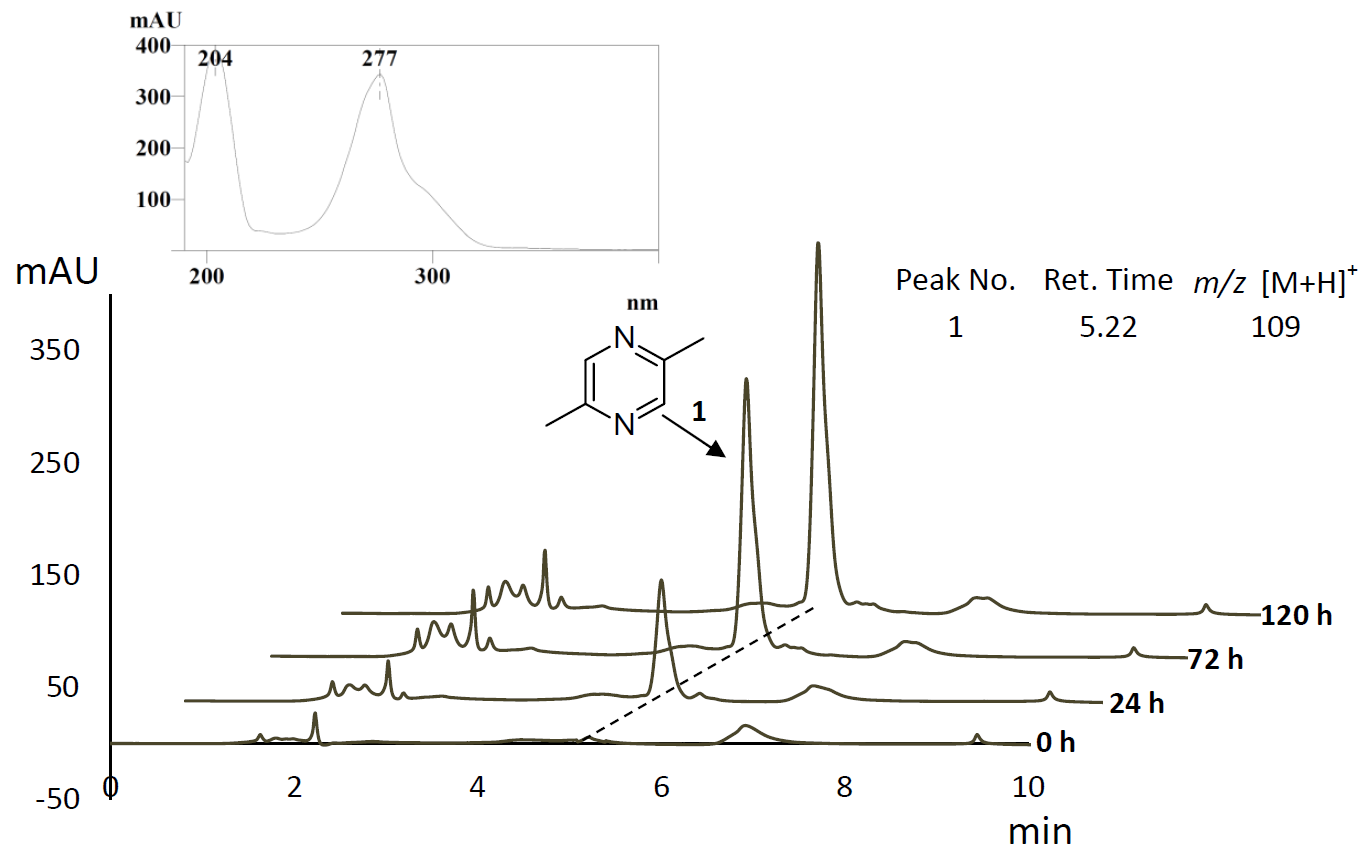


**Chromatogram S11.** A time-course of 2,5-DMP synthesis by *P. putida* KT2440Δ6 strain with pBAD2_tdh and pJNN _thrB_thrC_thrA^S345F^ plasmids, cultivated in MSM medium without the additional L-threonine (277 nm wavelength).


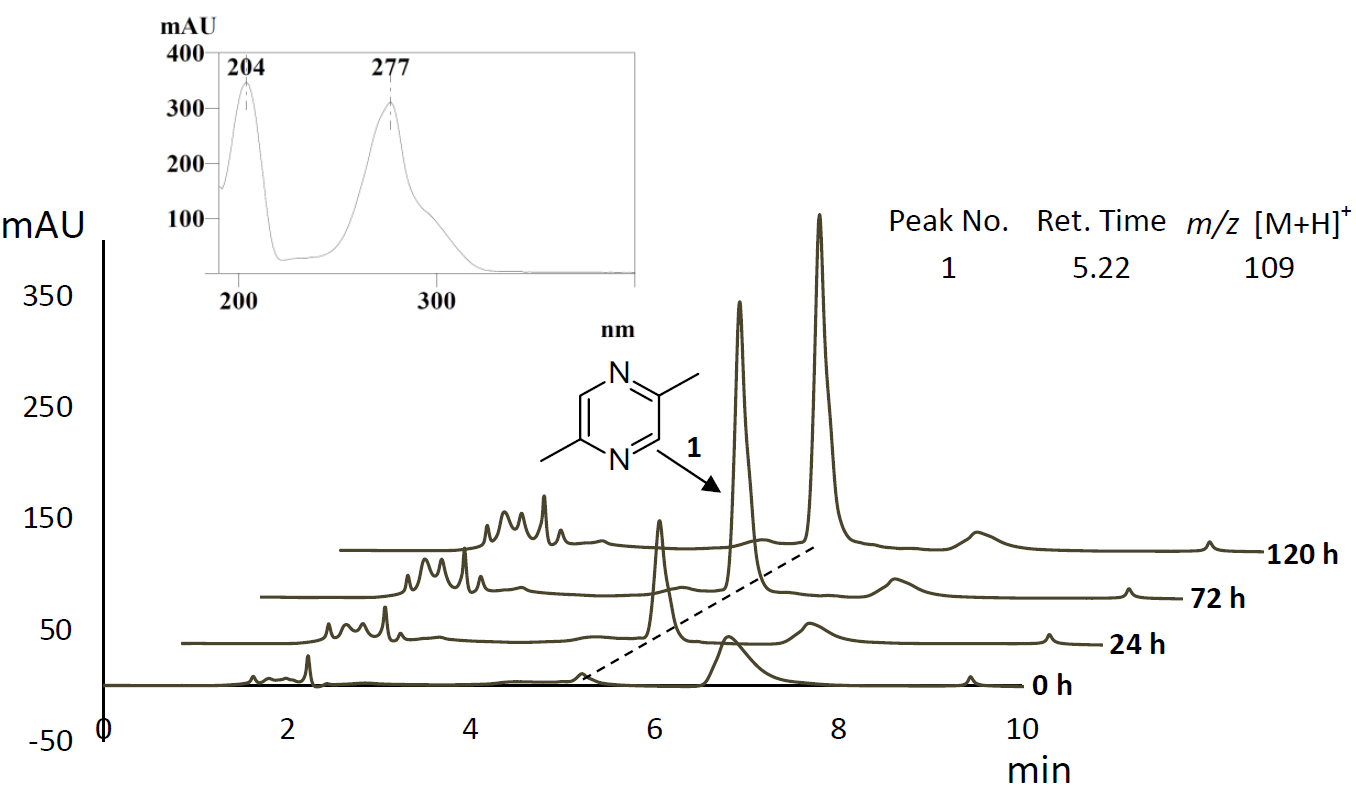


**Chromatogram S12.** A time-course of 2,5-DMP synthesis by *P. putida* KT2440Δ6 strain harboring pJNN_tdh_thrA^S345F^ and pBNT_thrB_thrC plasmids, cultivated in MSM medium without the additional L-threonine (277 nm wavelength).


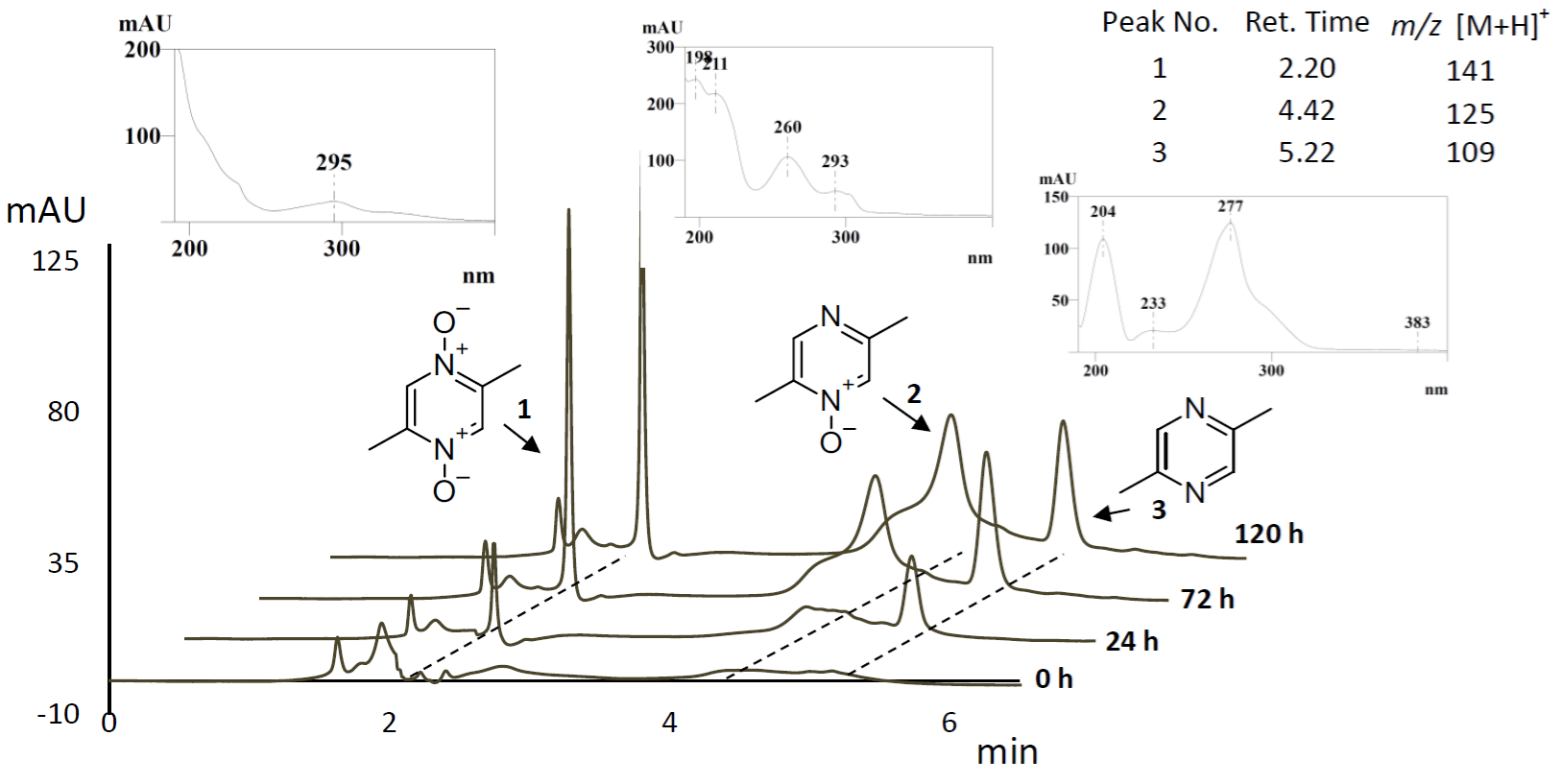


**Chromatogram S13.** A time-course of 2,5-DMP and N-oxides synthesis by *P. putida* KT2440Δ6 strain with pJNN_tdh_thrA^S345F^ and pBNT_Pml plasmids, cultivated in MSM medium without the additional L-threonine (300 nm wavelength). Induction of plasmids was executed simultaneously.


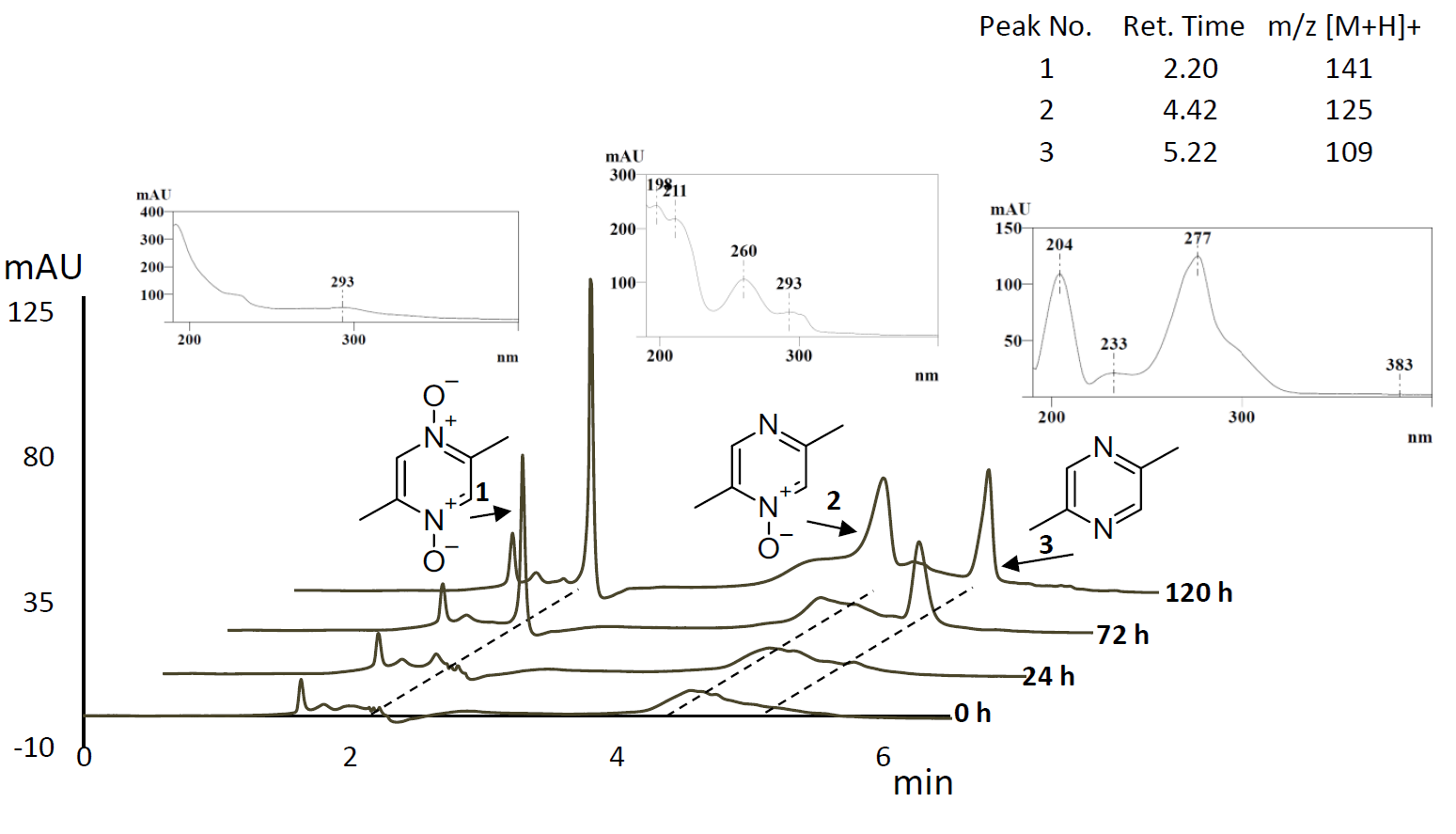


**Chromatogram S14.** A time-course of 2,5-DMP and N-oxides synthesis by *P. putida* KT2440Δ6 strain with pJNN_tdh_thrA^S345F^ and pBAD_Pml plasmids, cultivated in MSM medium without the additional L-threonine (300 nm wavelength). First, pBAD_Pml was induced, followed by the induction of pJNN_tdh_thrA^S345F^ 16 hours later.


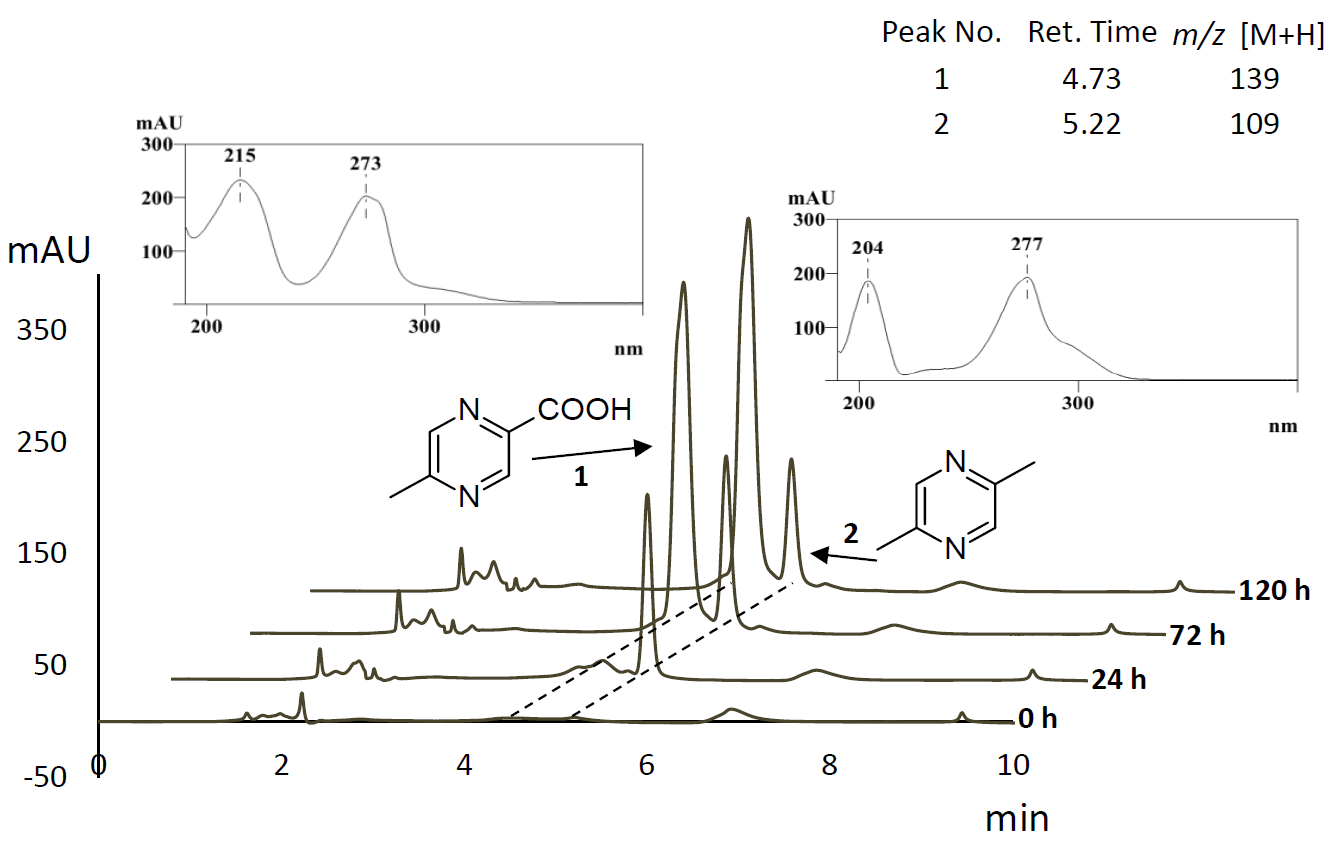


**Chromatogram S15.** A time-course of 2,5-DMP and MCPA synthesis by *P. putida* KT2440Δ6 strain with pJNN_tdh_thrA^S345F^ and pBNT_XylMABC plasmids, cultivated in MSM medium without the additional L-threonine (273 nm wavelength). Induction of plasmids was executed simultaneously.


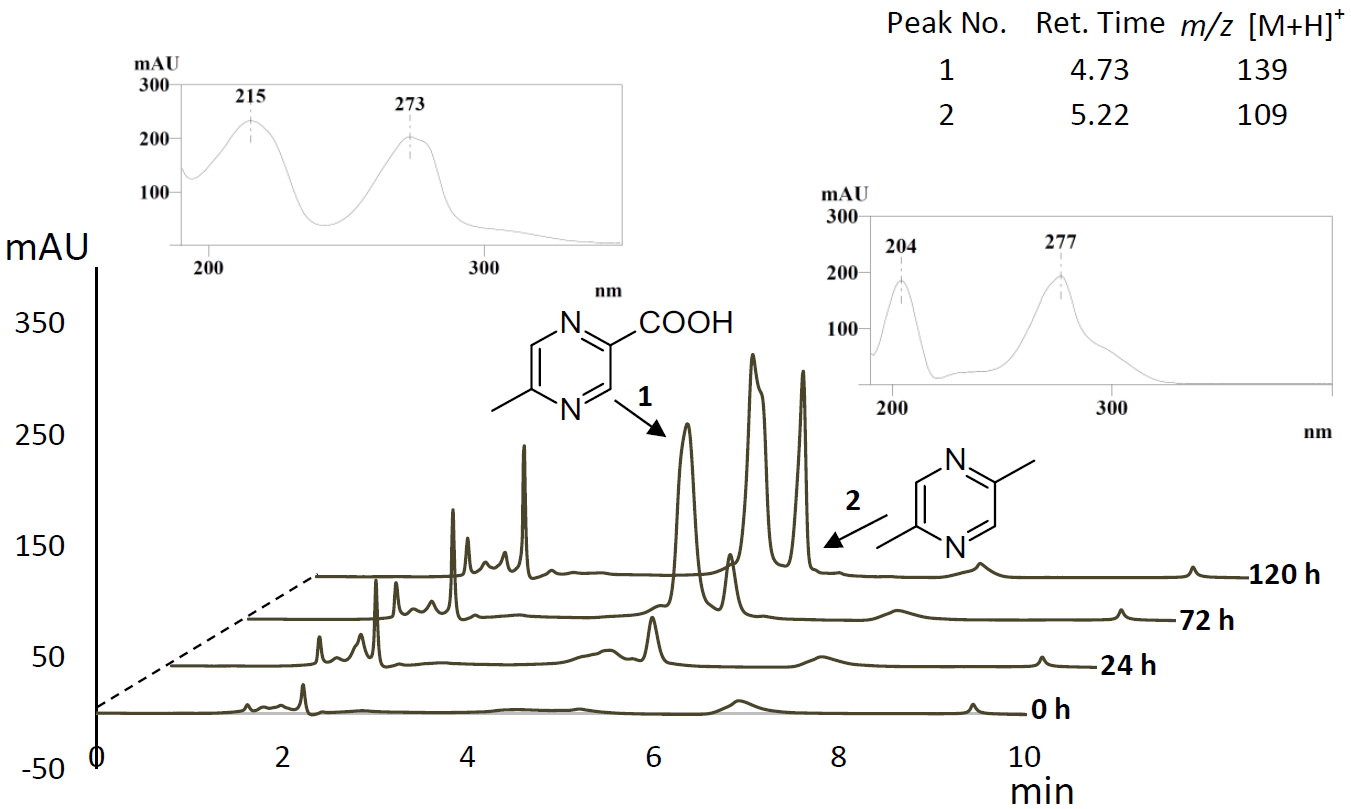


**Chromatogram S16.** A time-course of 2,5-DMP and MCPA synthesis by *P. putida* KT2440Δ6 strain with pJNN_tdh_thrA^S345F^ and pBAD_XylMABC plasmids, cultivated in MSM medium without the additional L-threonine (273 nm wavelength). First, pBAD_XylMABC was induced, followed by the induction of pJNN_tdh_thrA^S345F^ 16 hours later.


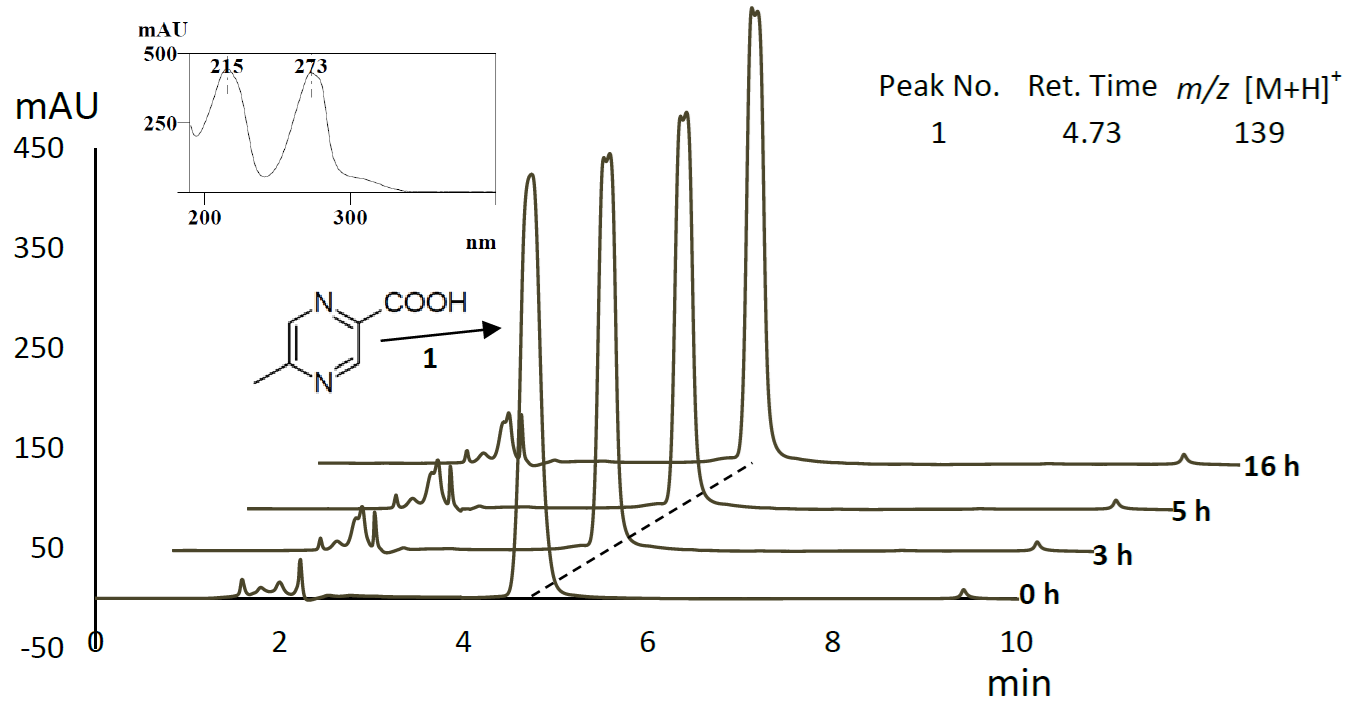


**Chromatogram S17.** A time-course of MCPA bioconversion by *P. putida* KT2440Δ6 whole-cells harboring pBAD_Pml plasmid (273 nm wavelength). Reaction conditions were as follows: 4 mM of MCPA, 10 g/L CDW, 30 ˚C, reaction volume of 5 mL.


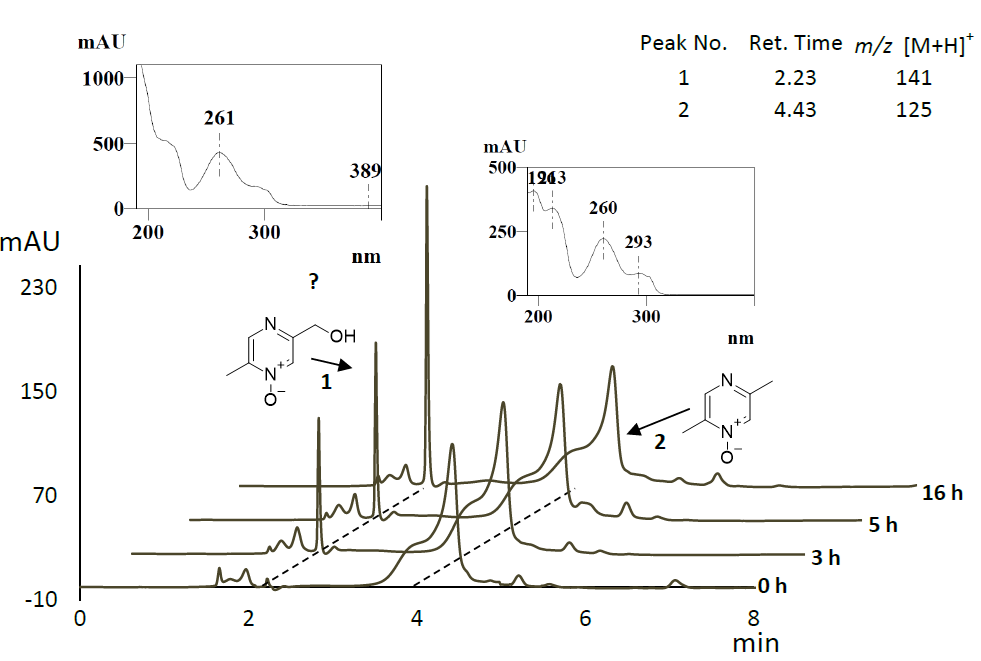


**Chromatogram S18.** A time-course of 2,5-DMP-N-OX bioconversion by *P. putida* KT2440Δ6 whole-cells harboring pBAD_XylMABC plasmid (280 nm wavelength). Reaction conditions were as follows: 2 mM of 2,5-DMP-N-OX, 10 g/L CDW, 30 ˚C, reaction volume of 5 mL.

# 1H and 13C NMR spectra of pyrazine products

2,5-dimethylpyrazine 1-oxide (2,5-DMP-N-oxide) obtained as a white solid. ^1^H BMR (400 MHz, DMSO-d_6_): δ = 2,28 (s, 3H, CH), 2,38 (s, 3H, CH), 8,30 (s, 1H, CH), 8,47 (s, 1H, CH). ^13^C BMR (101 MHz, DMSO-d_6_): δ = 13.98, 21.13, 132.34, 140.92, 147.10, 155.19.


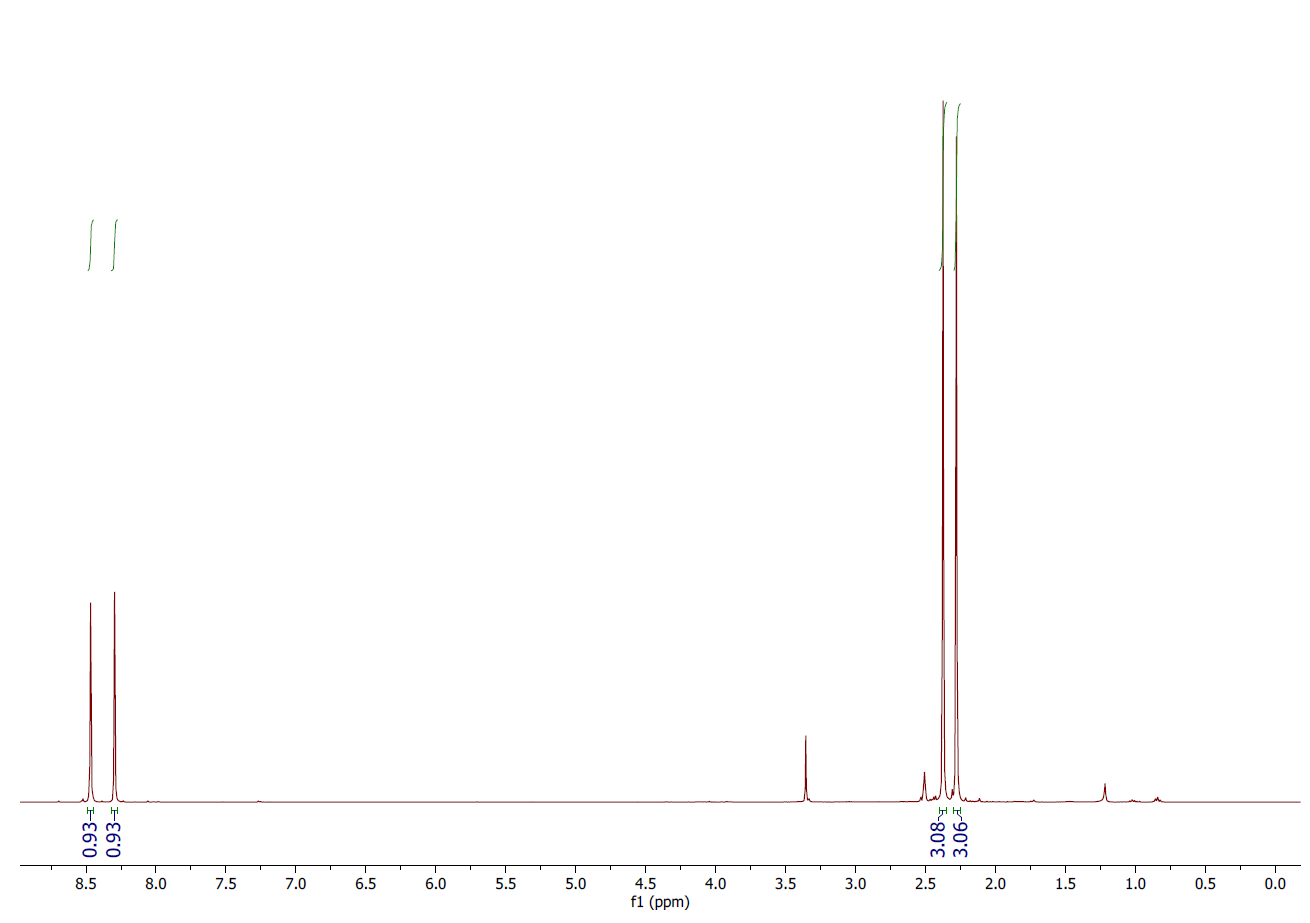


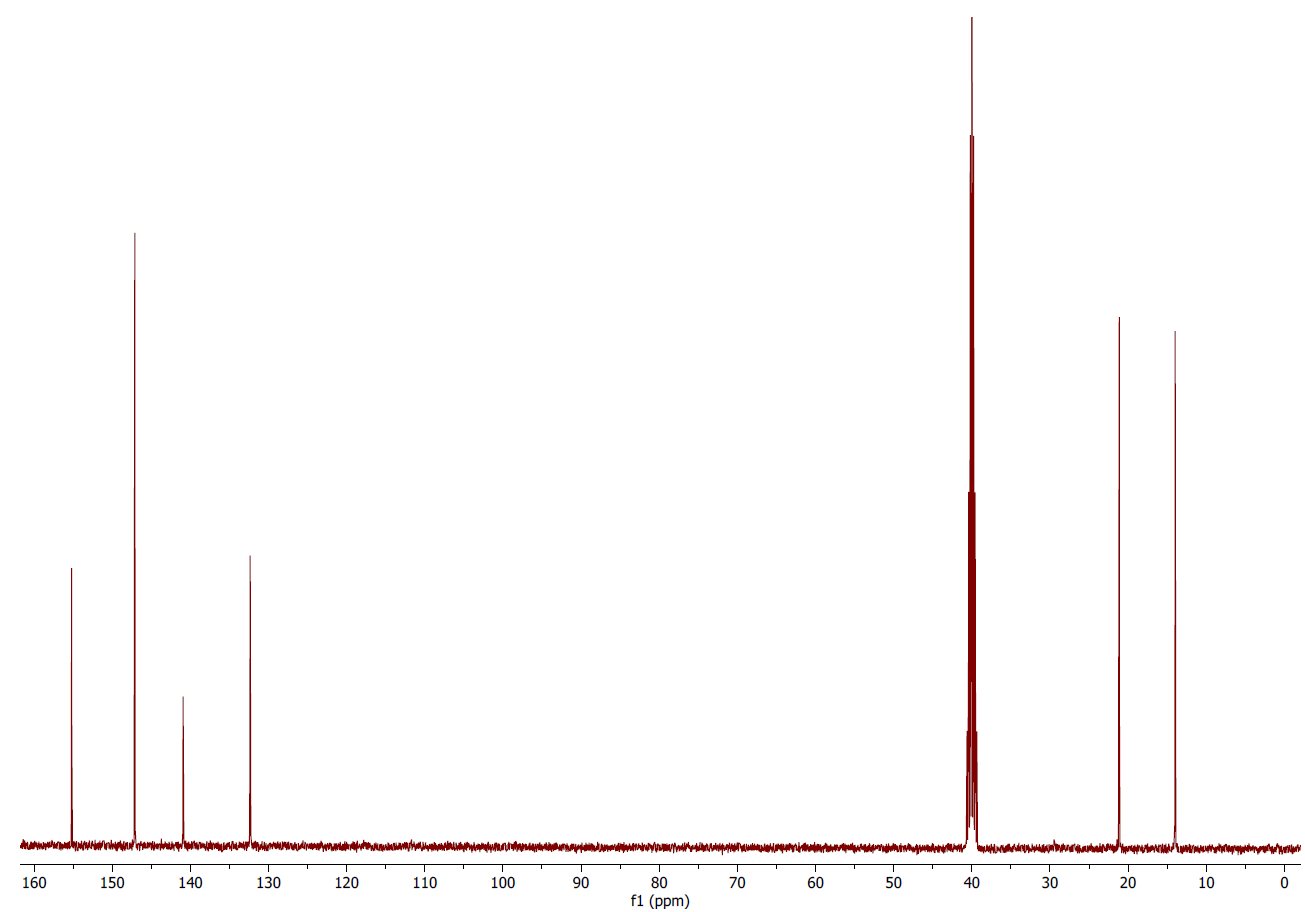


2,5-dimethylpyrazine 1,4-dioxide (2,5-DMP-di-OX) was obtained as a yellowish solid. ^1^H BMR (400 MHz, DMSO-d6): δ = 2,23 (s, 6H, CH), 8,50 (s, 2H, CH). ^13^C BMR (101 MHz, DMSO-d6): δ = 14.57, 135.24, 144.41


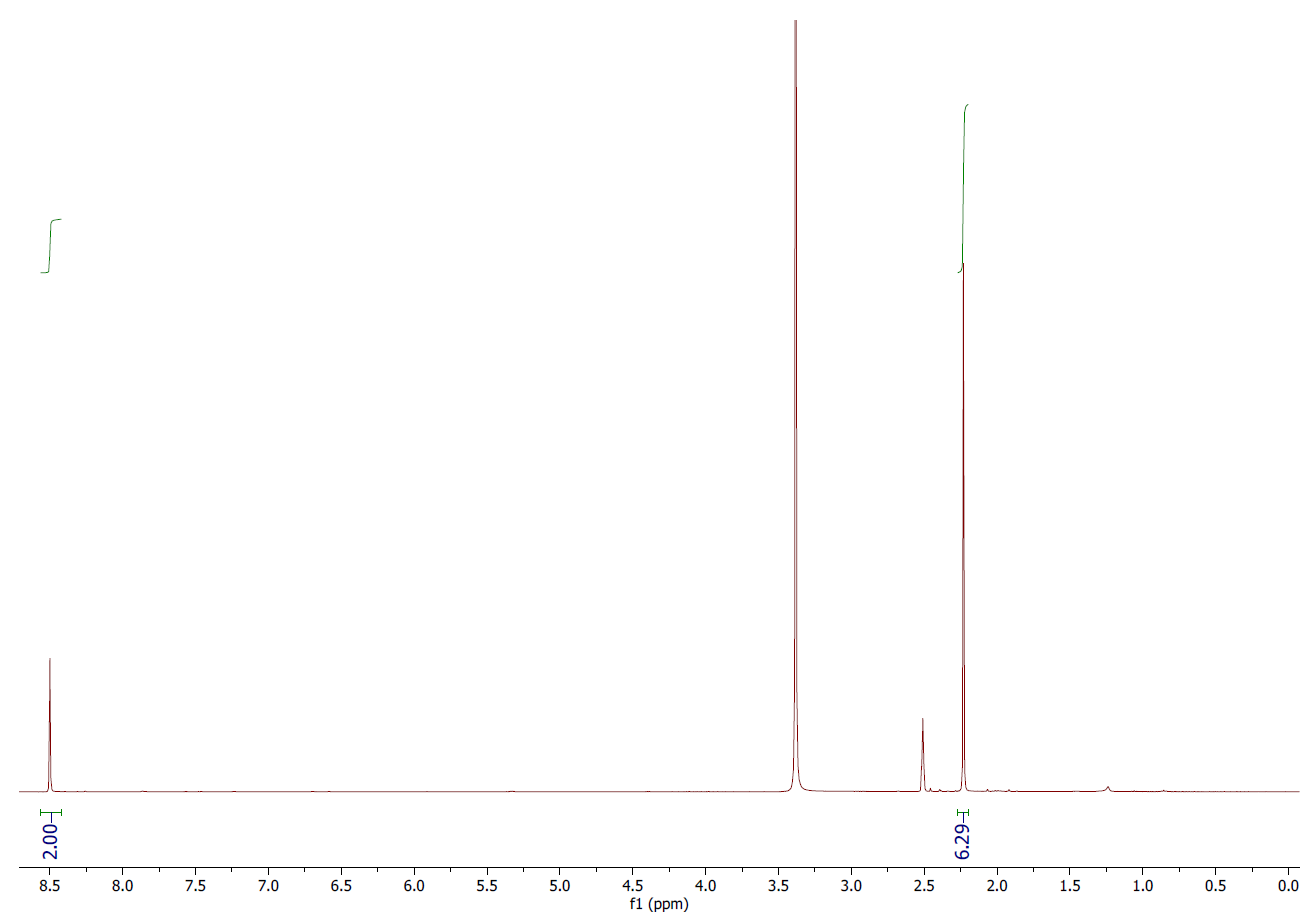


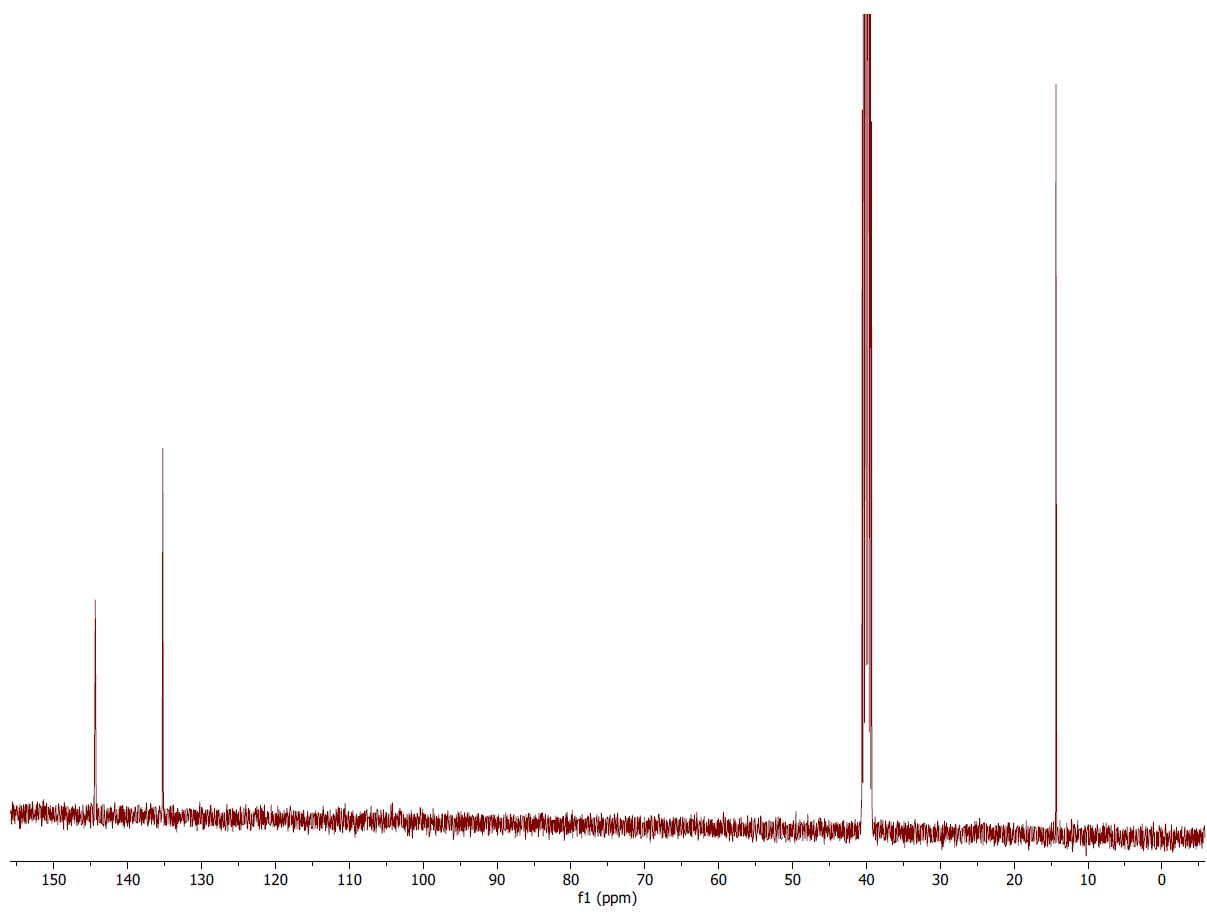


5-methyl-2-pyrazinecarboxylic acid (MPCA) was isolated as a pale grey solid. ^1^H BMR (400 MHz, DMSO-d6): δ = 2,59 (s, 3H, CH), 8.68 (d, *J* = 1.5 Hz, 1H, CH), 9.06 (d, *J* = 1.4 Hz, 1H, CH). ^13^C BMR (101 MHz, DMSO-d6): δ = 21.88, 141.46, 145.09, 144.75, 157.78, 165.75.


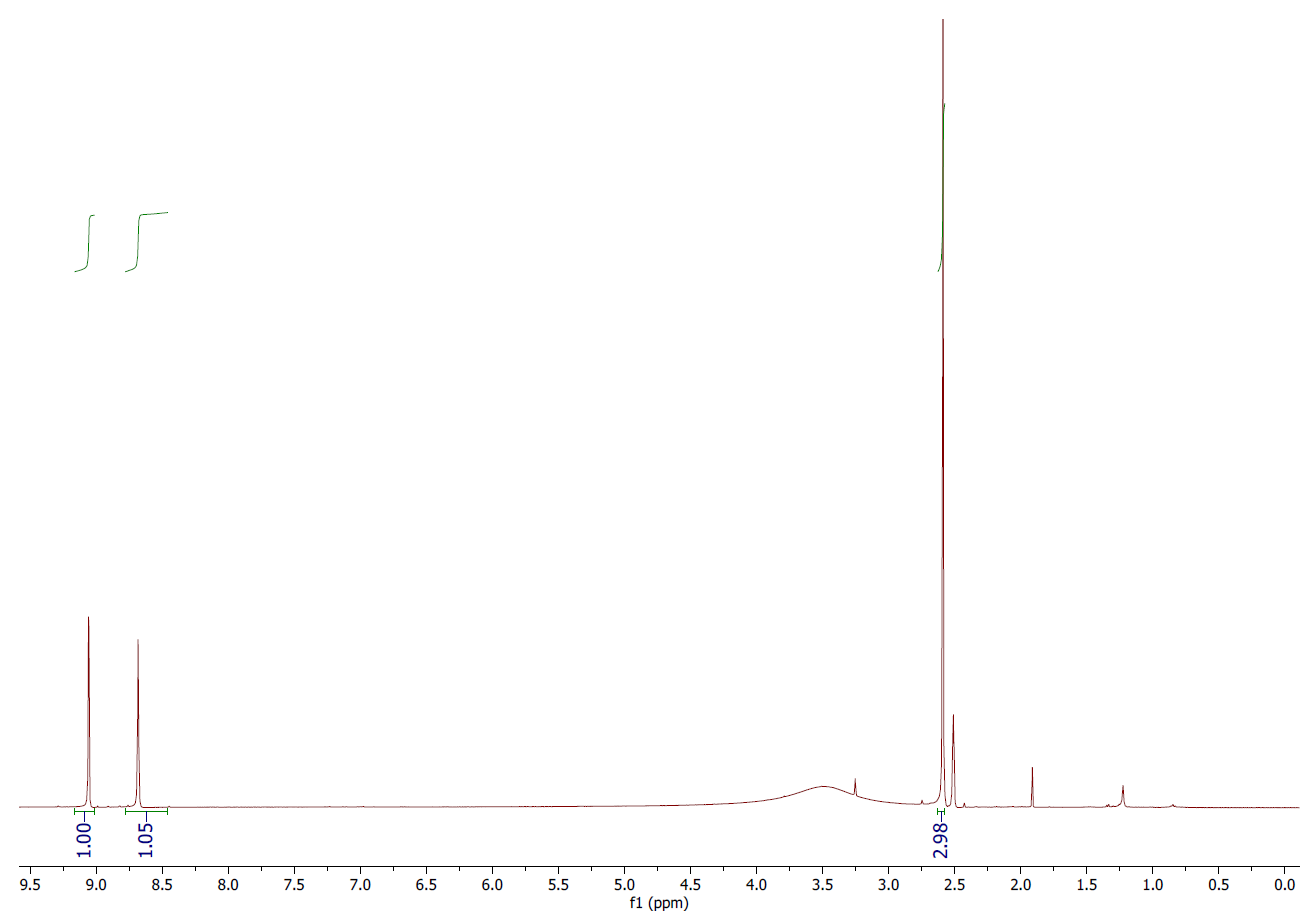


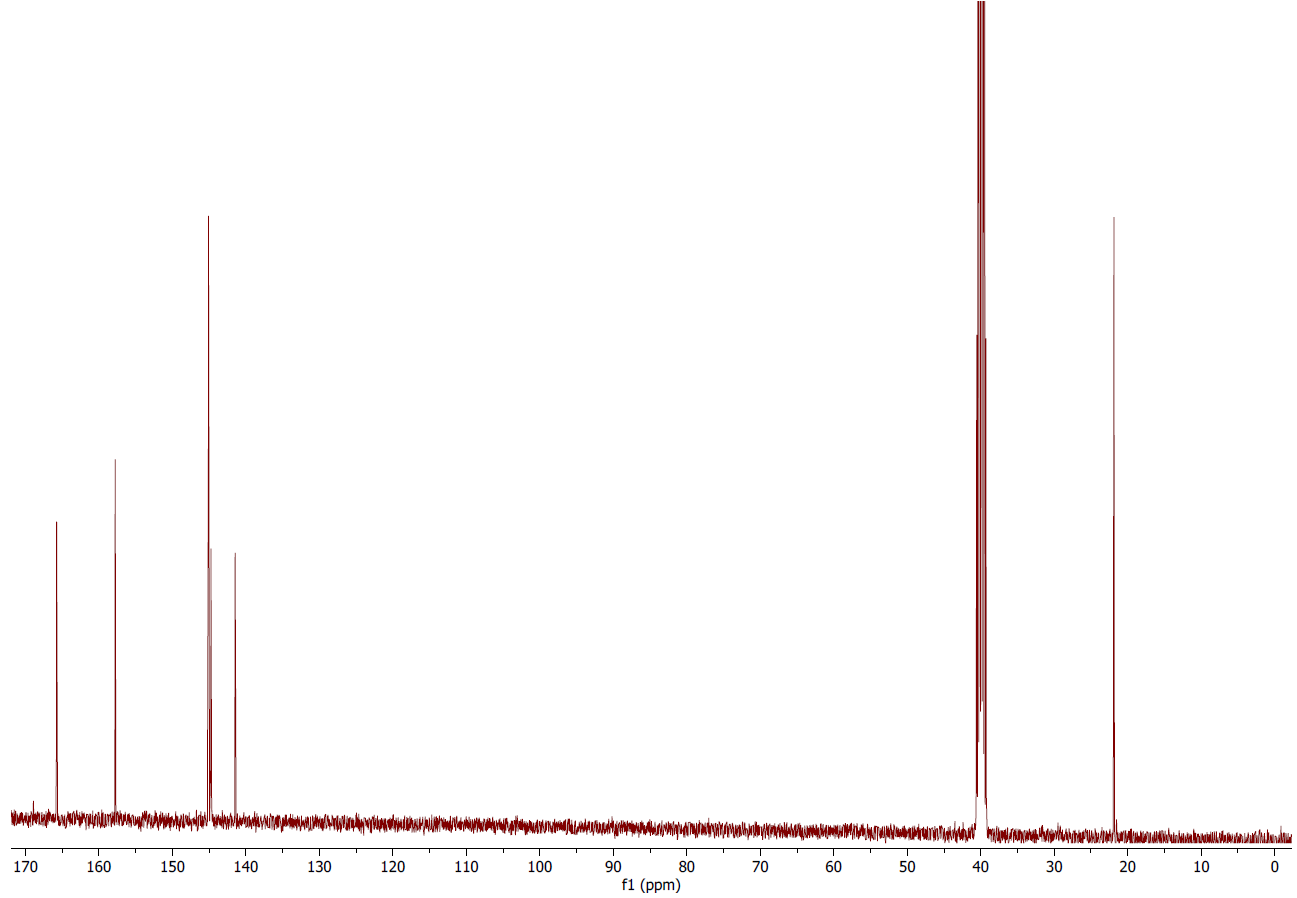

Supplement: Multimedia component 1 [file mmc1.docx]
